# Human motor cortical beta bursts relate to movement planning and response errors

**DOI:** 10.1371/journal.pbio.3000479

**Published:** 2019-10-04

**Authors:** Simon Little, James Bonaiuto, Gareth Barnes, Sven Bestmann

**Affiliations:** 1 Department of Clinical and Movement Neuroscience, UCL Queen Square Institute of Neurology, London, United Kingdom; 2 Department of Neurology, University of San Francisco, California, United States of America; 3 Wellcome Centre for Human Neuroimaging, UCL Queen Square Institute of Neurology, London, United Kingdom; 4 Institut des Sciences Cognitives Marc Jeannerod, CNRS UMR 5229, Bron, France; 5 Université Claude Bernard Lyon I, Lyon, France; UCSF, UNITED STATES

## Abstract

Motor cortical beta activity (13–30 Hz) is a hallmark signature of healthy and pathological movement, but its behavioural relevance remains unclear. Using high-precision magnetoencephalography (MEG), we show that during the classical event-related desynchronisation (ERD) and event-related synchronisation (ERS) periods, motor cortical beta activity in individual trials (*n* > 12,000) is dominated by high amplitude, transient, and infrequent bursts. Beta burst probability closely matched the trial-averaged beta amplitude in both the pre- and post-movement periods, but individual bursts were spatially more focal than the classical ERS peak. Furthermore, prior to movement (ERD period), beta burst timing was related to the degree of motor preparation, with later bursts resulting in delayed response times. Following movement (ERS period), the first beta burst was delayed by approximately 100 milliseconds when an incorrect response was made. Overall, beta burst timing was a stronger predictor of single trial behaviour than beta burst rate or single trial beta amplitude. This transient nature of motor cortical beta provides new constraints for theories of its role in information processing within and across cortical circuits, and its functional relevance for behaviour in both healthy and pathological movement.

## Abstract 2

The dominant cortical signal before and after movement is the beta oscillations over sensorimotor cortex, which are thought to slowly modulate around movement. This study shows that trial-by-trial beta signals are highly dynamic and dominated by short, high-power bursts, whose precise timing is linked to motor behavior.

## Introduction

Cortical activity in the beta frequency range (13–30 Hz) has been recognised for nearly a century, and occurs with systematic modulations before, during, and after movement [1–8].

Pre-movement beta is, on average, characterised by slow and spatially diffuse decreases in amplitude, which can start 1–2 seconds prior to movement onset [9]. This average pre-movement beta signal is commonly referred to as the event-related desynchronisation (ERD) and is influenced by a wide range of processes, including motor preparation [10–12], decisions for actions [13–15], and movement kinematics [3,6,7,16]. Following movement, average beta activity increases before gradually returning to baseline. This event-related synchronisation (ERS) has been linked to processes relating to inhibition of premature and unwanted responses [17–19], and updating of movement outcomes [20–24].

In addition to healthy movement control, understanding the functional role of cortical beta activity is vital to unlocking understanding of the pathophysiology of diseases of the human motor system [25–28]. However, despite being such a reliable hallmark neural signature of healthy and pathological movement, its association with a variety of processes including top-down communication [29–31], status quo maintenance [32], sensory sampling [33], motor stiffening [34], sensorimotor integration [33,35–37], and attention [38], arbitrating between different theories regarding the functional role of movement-related beta activity has been challenging. Analytical techniques that average beta activity within and across trials improve signal-to-noise ratio (SNR) but can disguise rapid signal fluctuations and their putative functional relevance.

Recent animal work and isolated reports in humans suggest, however, that slow, sustained changes in beta amplitude pre- and post-movement may not sufficiently summarise trial-wise dynamics in beta activity [39–43]. Specifically, work in the frontal cortex of nonhuman primates suggests that cortical beta activity is characterised by transient bursting that may only appear to be temporally sustained if averaged over multiple trials, with the highest probability of occurrence following movement, but with a wide temporal dispersion [42,44]. This observation is supported by recent work on the putative functional role of beta transients in sensory processing [45] and working memory [44,46]. In somatosensory cortex, beta bursting relates to tactile sensory discrimination, whereby non-detection is linked to higher burst rate and temporal coincidence with sensory input [45].

Trial-averaged beta activity may therefore provide a static summary of the underlying cortical burst rate, an idea supported by biophysical modelling of interlaminar neural dynamics [45,47,48]. Collectively, these findings suggest a prominent functional role for rapid beta bursts that may be partially concealed in conventional (between and within) trial-averaged analyses. Importantly, if average beta activity is indeed a static summary of underlying bursts dynamics, theories of its functional role need to incorporate this transient nature and its relevance for behaviour.

Despite the intimate role of motor beta activity in healthy and pathological movement, little is known in healthy human participants about its transient, burst-like nature and the behavioural relevance of such activity. We therefore here test the hypothesis that motor cortical beta may be dominated by burst activity, and that these bursts not only relate to behaviour but also have a stronger behavioural relationship than slowly modulated beta amplitude changes, as captured through conventional within- and across-trial averaging.

To this end, we characterise cortical beta bursts on a trial-by-trial basis, including their rate, size, and timing, before and after healthy human movement. Critically, we compare two alternative hypotheses. The first is that the functionally relevant signal is a sustained, amplitude-modulated beta signal and that beta bursts mainly represent noise and are unrelated to behaviour (H0). Our alternative hypothesis is that beta bursts dominate pre- and post-movement beta activity, with beta amplitude being an insufficient summary metric of burst occurrence that underestimates the relationship of beta to behaviour (H1). Evidence for H1 would require reinterpretation of the functional role of beta, based not on its amplitude but on burst rate and timing. Using human high-precision magnetoencephalography (MEG) recordings [49–51] and single trial behavioural modelling, we show that the precise timing of beta bursts in motor cortex relates closely to movement initiation and the commission of errors. This suggests that cortical beta bursts play an active, specific role in movement goal selection and updating, processes that are obscured by using commonly used, average analyses and interpretations based on sustained timescales.

## Results

**Fig 1:**
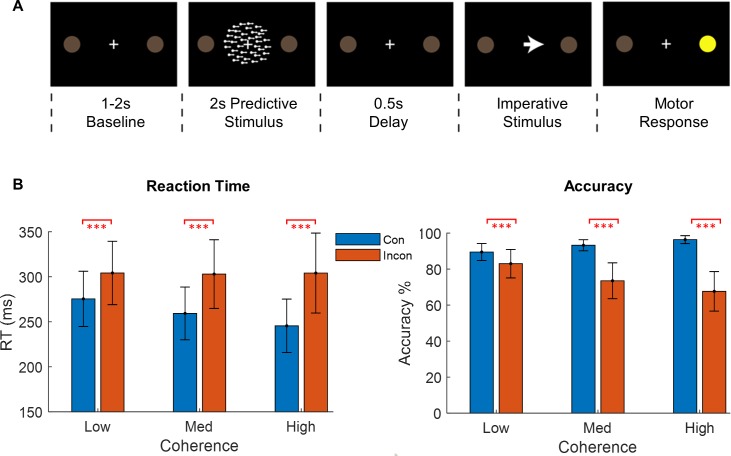
Task design and behavioural results. **(A)** Behavioural paradigm. Following baseline, a random dot kinematogram (RDK) indicates the likely direction of a subsequent (congruent or incongruent) imperative stimulus that signals a left or right button press using the index and middle fingers of the right hand. **(B)** Response times and accuracy across all subjects for all coherence levels and congruent (Con = blue) and incongruent (Incon = red) trials. Subjects responded more accurately and faster on congruent trials and were generally faster on these trials when the RDK motion coherence was strongest (red asterisks show all *p*-values < 0.001). The underlying data can be found at https://osf.io/eu6nx/. Med, medium; MEG, magnetoencephalography; RT, response time.

We recorded high SNR MEG using individual head-casts [49,50,52,53] in 8 healthy subjects during large numbers of trials in a probabilistically cued movement selection task (Fig 1A).

Across subjects, we recorded over 12,000 trials (per subject, M = 1,620; SD = 763.7) from multiple runs (1–3) per subject, repeated over several days (1–4) [51]. Within each 15-minute run, head movements were only 0.23 ± 0.04, 0.25 ± 0.05, and 0.99 ± 0.54 mm in the *x*, *y*, and *z* directions, respectively. The task was designed to vary the degree of movement preparation and subsequent speed of response. As such, the task involved a delay period in which a random dot kinematogram (RDK) informed subjects of the likely response indicated by a subsequent imperative stimulus (left or right; Fig 1A). The RDK had three levels of motion coherence, which manipulated the certainty with which the location of the imperative stimulus could be predicted. On 70% of trials, the imperative stimulus matched the direction of RDK (congruent), whereas in 30% of trials it signalled the opposite response (incongruent). This allowed for isolating movement preparation from initiation (as subjects could not commence movement until the imperative stimulus appeared, which indicated the required response) under different levels of preparedness. All subjects were able to complete the task, and the mean response time was 277.5 ± 29.6 milliseconds, with a mean error rate of 12.6 ± 4.7%.

Subjects responded more accurately and faster on congruent trials (Fig 1B), and were generally faster on these trials when the RDK motion coherence was strongest [51]. By contrast, subjects made more mistakes and were slower to respond on incongruent trials. The response times and accuracy were analysed using generalised linear mixed models (see Materials and methods), revealing an interaction between congruence and coherence for accuracy (χ^2^(2) = 377.02, *p* < 0.001) and response time (RT; F(2,12947) = 22.7, *p* < 0.001) (Fig 1B, S1 Table, see [51] for details).

Pairwise comparisons (Tukey corrected for multiple comparisons) showed that accuracy was higher and RTs were faster during congruent trials than incongruent trials at low (accuracy: Z = 8.23, *p* < 0.001; RT: t(12,939.87) = −7.58, *p* < 0.0001), medium (accuracy: Z = 23.82, *p* < 0.001; RT: t(12,939.87) = −13.63, *p* < 0.001), and high coherence levels (accuracy: Z = 29.59, *p* < 0.001; RT: t(12,939.87) = −17.0, *p* < 0.001). Subjects thus were more accurate and faster when visual cue information about the forthcoming action was more reliable.

### Motor cortical beta amplitude is highly dynamic in single trial data

**Fig 2:**
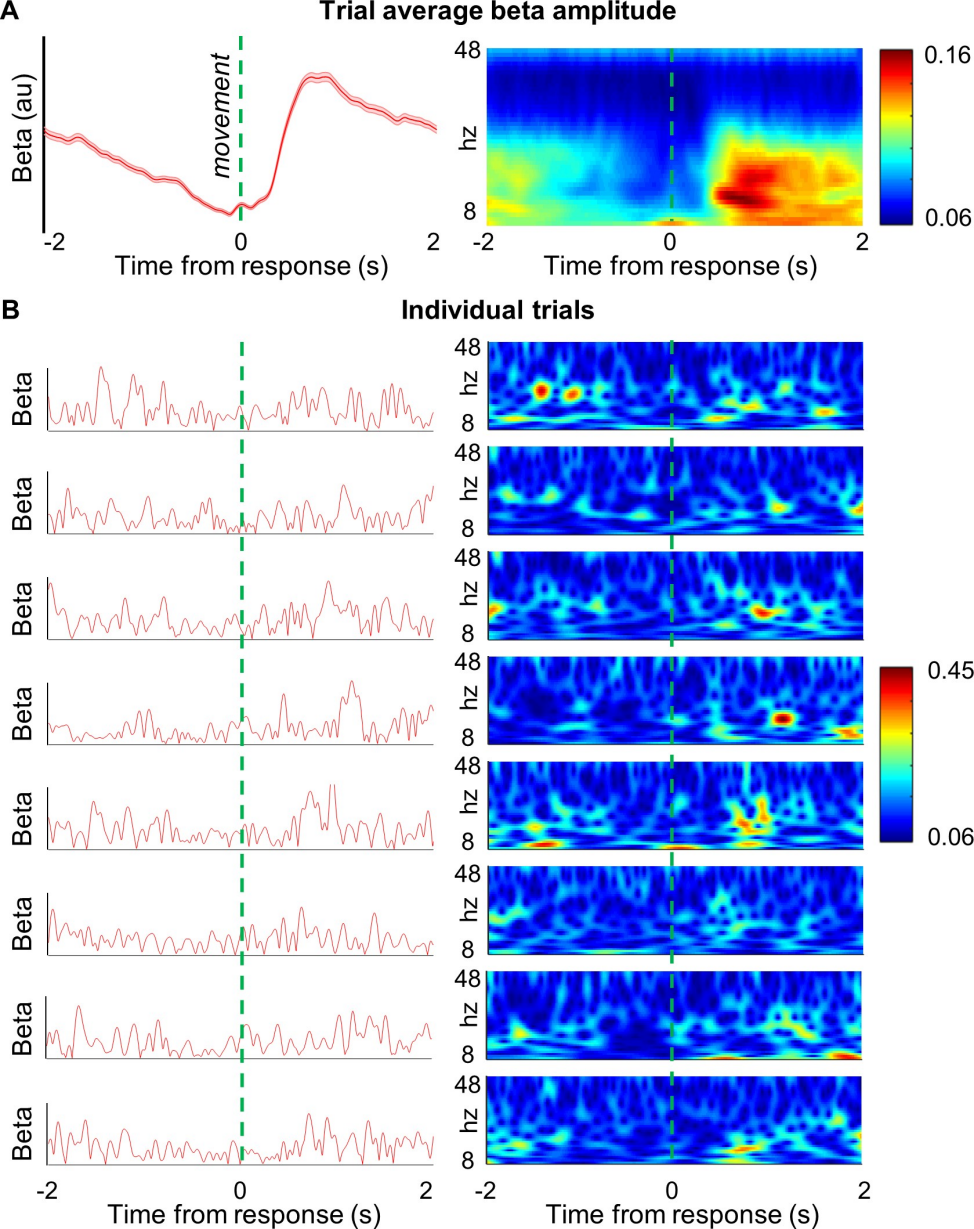
Single trial beta events do not match the dynamics of trial-averaged beta activity. **(A)** Time series of trial-averaged contralateral M1 beta amplitude (13–30 Hz; subject 2; block 1; mean ± SEM 535 trials; left panel). This shows classical ERD and ERS in the pre- and post-movement periods, respectively. Trial-average time-frequency amplitude spectrogram (same subject; wavelet decomposition; see Materials and methods; right panel) showing a reduction in spectral activity specific to the beta band in the period prior to movement, followed by an increase following movement. **(B)** Individual trial data from the same subject (no. 2; run 3; trials 250–258) as the beta amplitude envelope (red; left panels) and as time-frequency amplitude spectrograms (right panels). At the single trial level, beta amplitude does not closely resemble the trial average but appears highly dynamic with short lasting bursts, which are more common around the time of the average ERS. The underlying data can be found in the post-processed files (Subj2Run3.mat) at https://osf.io/eu6nx/. ERD, event-related desynchronisation; ERS, event-related synchronisation.

Conventionally, beta amplitude envelopes have been averaged over trials to remove and smooth out rapid signal fluctuations, with attention focused (and behavioural correlations made) on the resulting slowly changing, mean beta changes (Fig 2A). We here asked whether beta bursts can be seen at the single trial level in healthy human subjects (Fig 2B).

Source inversion and virtual electrode creation (see additional methods in S1 Text) at the primary motor cortex contralateral to finger movement produced continuous time series of the beta signal (13–30 Hz). These were epoched to either the imperative stimulus (−3 → 1.5 seconds) or the movement onset (button press; −2 → 2 seconds), respectively, for corresponding analyses.

As per convention, we use ‘ERD’ and ‘ERS’ to denote average (across trials) changes in the magnitude of the beta signal before and after movement, respectively. At the single trial level, we use ‘amplitude’ to refer to the time varying envelope of the beta signal (√power), and we use the word ‘bursts’ to refer to the periods when the beta amplitude is above our empirically defined threshold.

**Fig 3:**
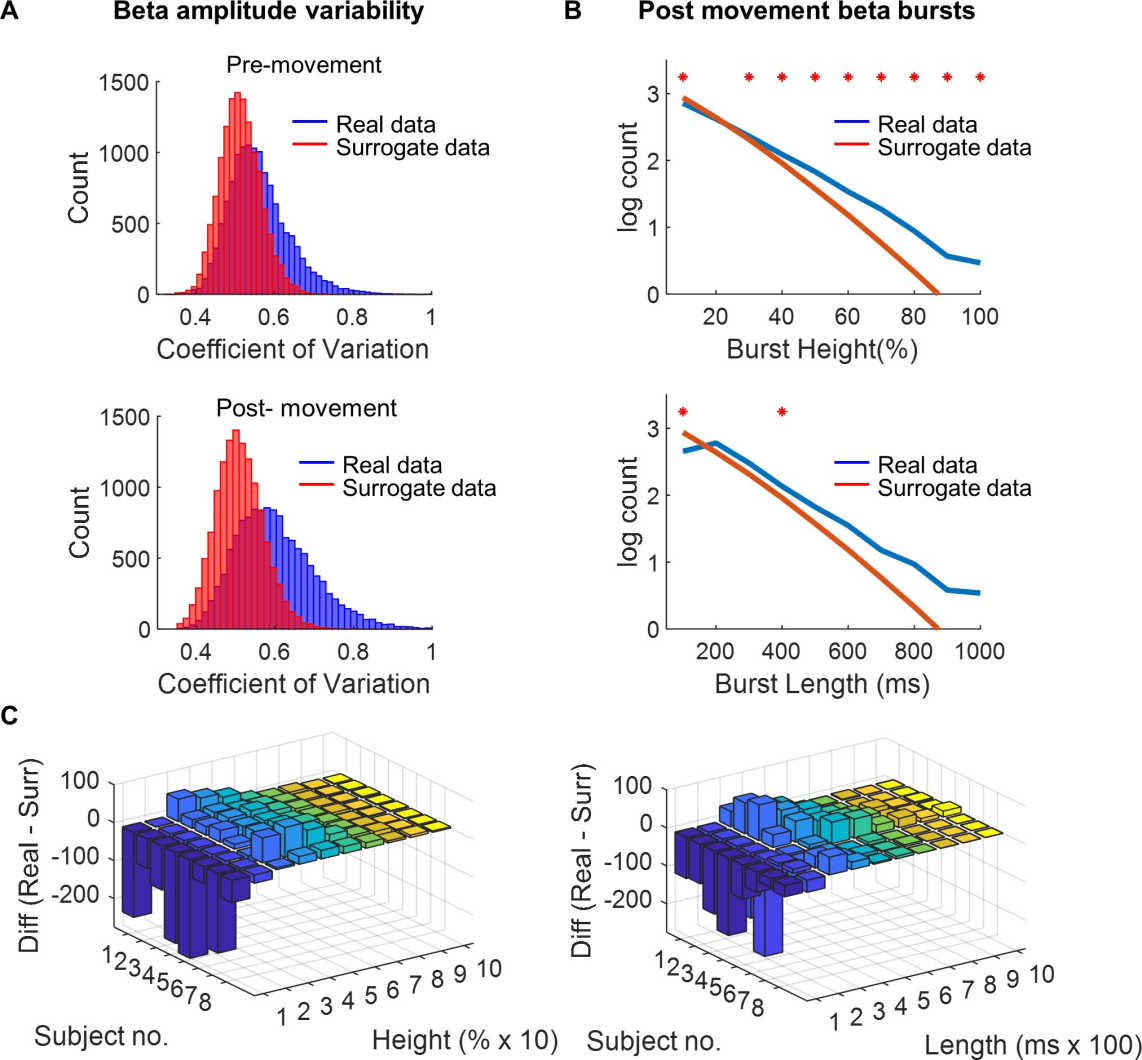
Comparison of movement-related beta amplitude changes and phase-randomised surrogate data. **(A)** Beta amplitude variability is quantified by the CV of beta amplitude for pre- and post-movement periods, respectively. The CV in the observed data is significantly larger (right shift in histogram) in both the pre- and post-movement periods when compared with a spectrally matched surrogate dataset. **(B)** Group-level line histogram of the log10 mean burst counts for different heights (B: top) and lengths (B: bottom) for real versus surrogate post-movement data (*p < 0.05, false discovery rate [FDR] corrected). **(C)** Difference in burst height and length counts shown for each subject compared with bursts from a surrogate phase-randomised dataset (burst height/length real data minus burst height/length surrogate data). This demonstrates a relative reduction in the number of the smallest and shortest bursts in the real compared with surrogate data, with an increase in larger and mid-length bursts (same analysis shown for pre-movement data in S2 Fig). The underlying data can be found at https://osf.io/eu6nx/. CV, coefficient of variation.

We first addressed whether the variance of the beta amplitude within the trial was the same as a null distribution of time series with the same power spectrum, implying that amplitude variance (bursts) may just be noisy events not indicative of behaviour (H0). Alternatively, we looked at whether this amplitude variance was increased (relative to our null distribution), signifying that it may be indicative of behavioural processes (H1). To address this, we compared the coefficient of variation (CV; amplitude normalised metric of variability = standard deviation / mean) of the beta amplitude in our data to a surrogate dataset with an identical amplitude distribution but randomised Fourier phase coefficients. The latter dataset retains the same overall amplitude characteristics as the original data but randomises how this spectral activity is presented over time (individual sinusoids phase-randomised in the frequency domain; see Material and methods). This creates the null distribution of the many possible time series that can create the power spectral density (PSD) found in the real data. We found that the CV of beta amplitude was larger in the observed data than would be expected by chance from the phase-randomised surrogate dataset. This was observed for both the pre-movement (*t*(7) = 21.3; *p* < 0.0001; Fig 3A) and post-movement periods (*t*(7) = 26.4; *p* < 0.0001; Fig 3A), respectively [42,54,55].

Previous work suggests that large amplitude beta bursts might be a behaviourally relevant component of beta activity [42,45,47,56]. We next directly compared the largest beta bursts in our observed data versus the phase-randomised surrogate dataset. We therefore first empirically determined in each individual, separately for the ERD and ERS periods, a threshold for identifying beta bursts, by correlating per-trial burst rate with beta amplitude (see Materials and methods [47]). Based on this empirically defined threshold of 1.75 standard deviations above the median, the top 11.9% or 12.8% of beta amplitude of the data in the pre-movement and post-movement periods, respectively, were defined as burst activity.

Having empirically determined a threshold that define bursts, we next compared bursts in the original dataset with those in the surrogate, spectrally matched but phase-randomised dataset. We found that there were a greater number of larger bursts in the observed data compared with the surrogate dataset (paired *t* tests at each data point; red asterisks all *p*_*FDR*_ < 0.05, Fig 3B and 3C; S2 Fig) for both pre- and post-movement. Burst duration was also longer in the observed than in the surrogate dataset (paired *t* tests at each data point; red asterisks all *p*_*FDR*_ < 0.05, Fig 3B and 3C) post-movement. Because pre- and post-movement epochs exhibit vastly different average amplitude levels, we addressed the possibility of different burst characteristics pre- and post-movement by examining average burst height and length in these two periods separately. Bursts were smaller in amplitude (*t*(7) = 2.3; *p* = 0.049) but showed no difference in duration (*t*(7) = −0.7; *p* = 0.49) in the pre- (96.2 ± 8.8 milliseconds) versus post-movement (94.2 ± 10.4 milliseconds) periods, respectively.

High amplitude beta bursts are therefore larger in amplitude and longer lasting than expected from phase-randomised surrogate data, i.e., than a random set of time series that all have the same spectral properties. This may suggest that high amplitude beta burst activity (as found relative to phase-randomised surrogate data) may be a functionally relevant signal rather than simply noise [57,58].

**Fig 4:**
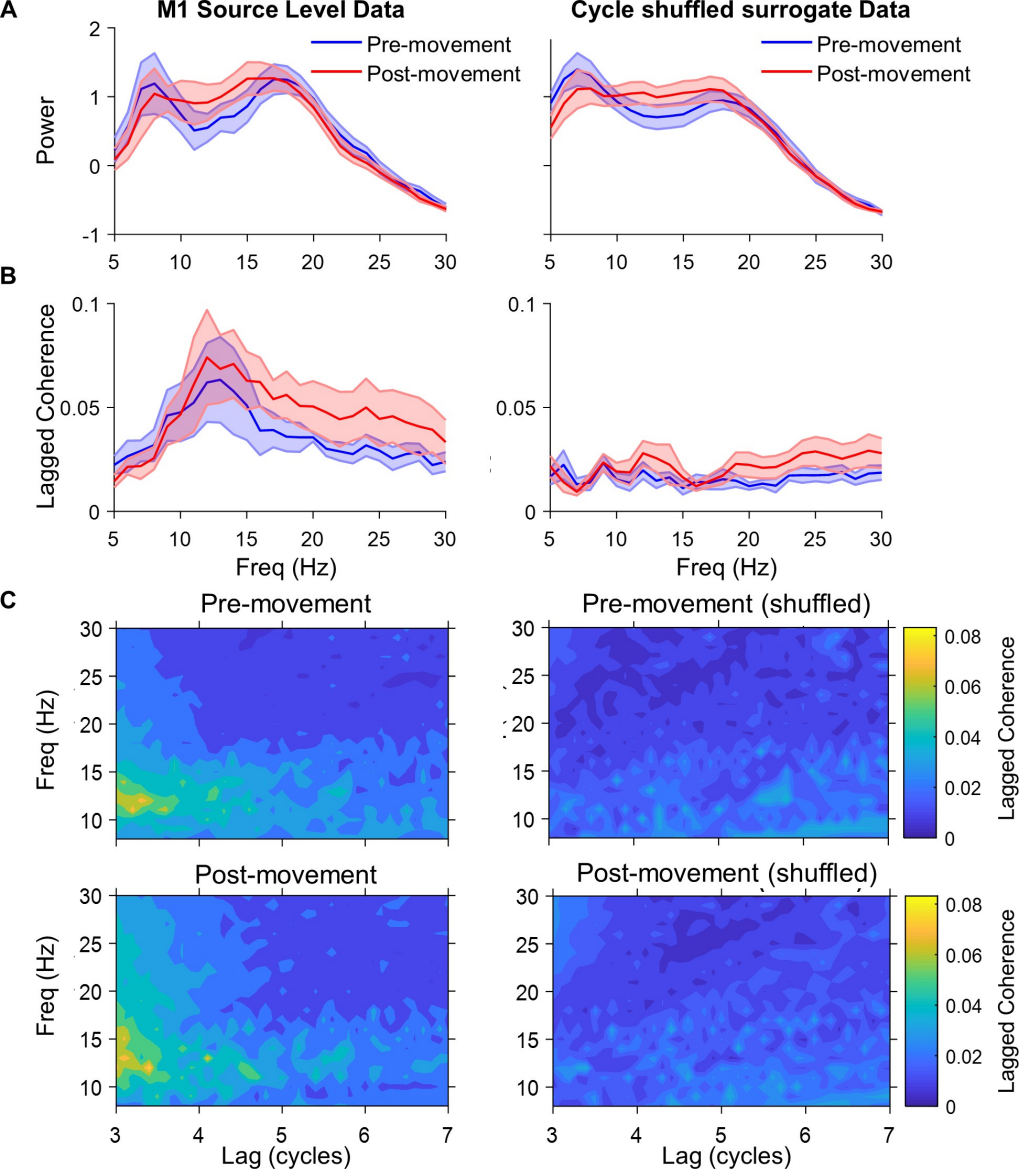
Motor cortical phase lagged coherence. **(A)** Mean PSD for all subjects (±SEM) for a virtual electrode from M1 (hand knob), response-locked data, with 1/f noise component removed (5–30 Hz). The power profile is preserved in the surrogate, cycle-shuffled data (right panel). Data shown both pre- and post- movement. **(B)** Lagged coherence (3 cycles) for real data (left) and cycle-shuffled surrogate control data (right). Note the absence of a strong peak in lagged coherence at the beta power peak of 18 Hz. **(C)** Lagged coherence for beta activity (13–30 Hz) rapidly falls off further after 3 cycles, compatible with burst activity (here shown for both pre-movement [top] and post-movement [bottom]). The underlying data can be found at https://osf.io/eu6nx/. PSD, power spectral density.

We next conducted another control analysis, which looked for evidence of sustained (amplitude-modulated) oscillatory beta activity in addition to the identified bursts [57,59]. One possibility is that beta activity is rhythmic and sustained but amplitude-modulated. In this case, the phase of beta in the present cycle should predict the phase of beta in the future, and this should extend over several cycles [57]. By contrast, if beta is dominated by burst activity without a significant additional amplitude-modulated component, its phase should only be predictable over a relatively short number of cycles. To compare these two alternatives, we computed the lagged coherence metric [59], a frequency-specific measure of the consistency between a signal’s current and future phases. Pre- and post-movement source space (M1) PSD and lagged coherence are presented for all frequencies (Fig 4). This analysis confirmed a peak in the spectrum in the beta range (Fig 4A). However, corresponding lagged coherence within the beta range in both the pre- and post-movement epochs fell rapidly off after just 3 cycles (<0.01; [57,59]). This finding is compatible with bursting activity but less in support of the idea of sustained oscillatory beta activity. A control analysis with an additional surrogate dataset (cycle shuffled; see Materials and methods) that preserved the power spectra but shuffled cycles in the time domain revealed much lower lagged coherence throughout all frequencies, suggesting an absence of short periods (bursts) of oscillatory activity in the cycle-shuffled surrogate data.

This, in addition to the phase-randomised surrogate data analysis and correlation between burst probability and conventional ERD/ERS, provides evidence that cortical beta in motor cortex may be dominated by bursts of activity, which would mask any weakly rhythmic, sustained, underlying signal.

### The distribution of beta bursts matches the time course of the ERD and ERS

We next examined the relationship between the occurrence of bursts (as quantified by burst probability over trials) and the classical trial-averaged beta amplitude signals before and after movement (ERD and ERS).

**Fig 5:**
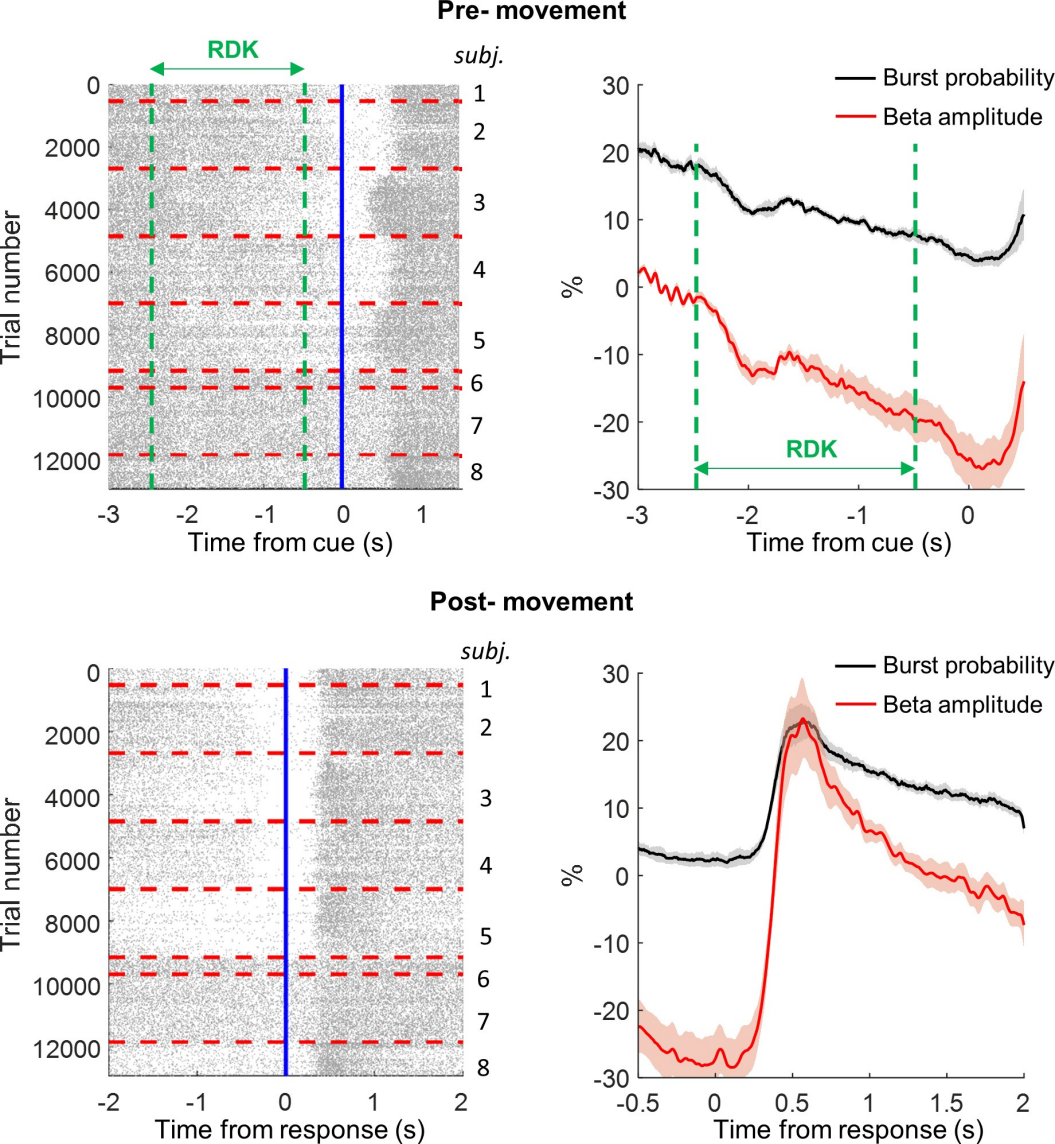
Trial-averaged beta amplitude of ERD and ERS is mirrored by burst probability across trials. Raster plots showing the timing of each individual burst (single dash = peak of burst) for all trials of all 8 subjects (>12,000 trials; individual subjects divided by dashed red lines). Top left panel: raster plot of beta bursts, locked to the imperative stimulus. Lower left panel: raster plot of beta bursts, locked to movement response. There is a consistent relationship of burst timing to movement onset, with a gradual reduction in burst probability in the time building up to movement, followed by a significant increase (weakly temporally locked) in burst probability after movement. Top right panel: normalised (percent versus pre-movement preparation [500-millisecond] baseline) average ERD ± SEM (red) is shown in the pre-movement period, with burst probability (%) overlaid (black). Lower right panel: same analysis for the post-movement ERS period, using the same baseline. The classical event-related desynchronisation (‘ERD’) and event-related synchronisation (‘ERS’) are shown in the pre- and post-movement periods, respectively, and mirrored in shape by the across-trial burst probability. The underlying data can be found at https://osf.io/eu6nx/. ERD, event-related desynchronisation; ERS, event-related synchronisation; RDK, random dot kinematogram.

The average beta burst rate for the whole pre-movement period was 1.3 ± 0.08 bursts per second (−3 seconds to 0 seconds prior to imperative stimulus). However, these bursts were not evenly distributed, as shown by the trial burst distribution over time (Fig 5). Burst probability gradually declines prior to movement onset (Fig 5). During the pre-movement period, the baseline (500 milliseconds before trial onset) normalised classical average beta amplitude across trials (percent relative to the 500-millisecond period before preparatory RDK baseline) decreased from −2.3 ± 0.7% below baseline (−2.5 seconds prior to the imperative stimulus presentation) to −25.5 ± 3.7% below baseline (at the imperative stimulus—equivalent to classical ERD; Fig 5). During the same pre-movement period, the beta burst probability (the probability of beta amplitude being over the threshold at any given time point, across all trials) decreased from 20.3 ± 0.7% to 4.5 ± 1.0% at the imperative stimulus (top right panel Fig 5).

Post-movement, the average beta burst rate was 1.51 ± 0.08 bursts per second. When normalised to baseline (percent relative to the 500-millisecond period before preparatory RDK baseline), the average beta signal increased from −27.2 ± 4.0% at button press to 23.2 ± 6.1% at the group-level peak of beta (equivalent to the classical ERS; 568 milliseconds). During this period, the beta burst probability increased from 2.3 ± 1.0% to 22.9 ± 2.5% (right lower panel Fig 5B). Therefore, even at the peak of the ERS, bursts occurred infrequently, with different numbers of bursts occurring on individual trials during this period (<1 in 4 trials at this precise moment). Finally, we correlated the burst probability across trials (black line Fig 5B) with average beta amplitude across trials (red line Fig 5B) for each subject to determine how changes in beta bursting probability tracked the conventional average amplitude signal. Notably, in all participants, the trial average beta amplitude in the ERD and ERS period were closely correlated with the burst probability (mean *R*^2^ of 0.93 ± 0.02 [ERD]; mean R^2^ of 0.93 ± 0.03 [ERS]). This confirms that the burst probability across trials could account for the majority of variance in the classical ERD and ERS signals.

### Beta bursts are spatially more focal than averaged beta activity during the ERS period

**Fig 6:**
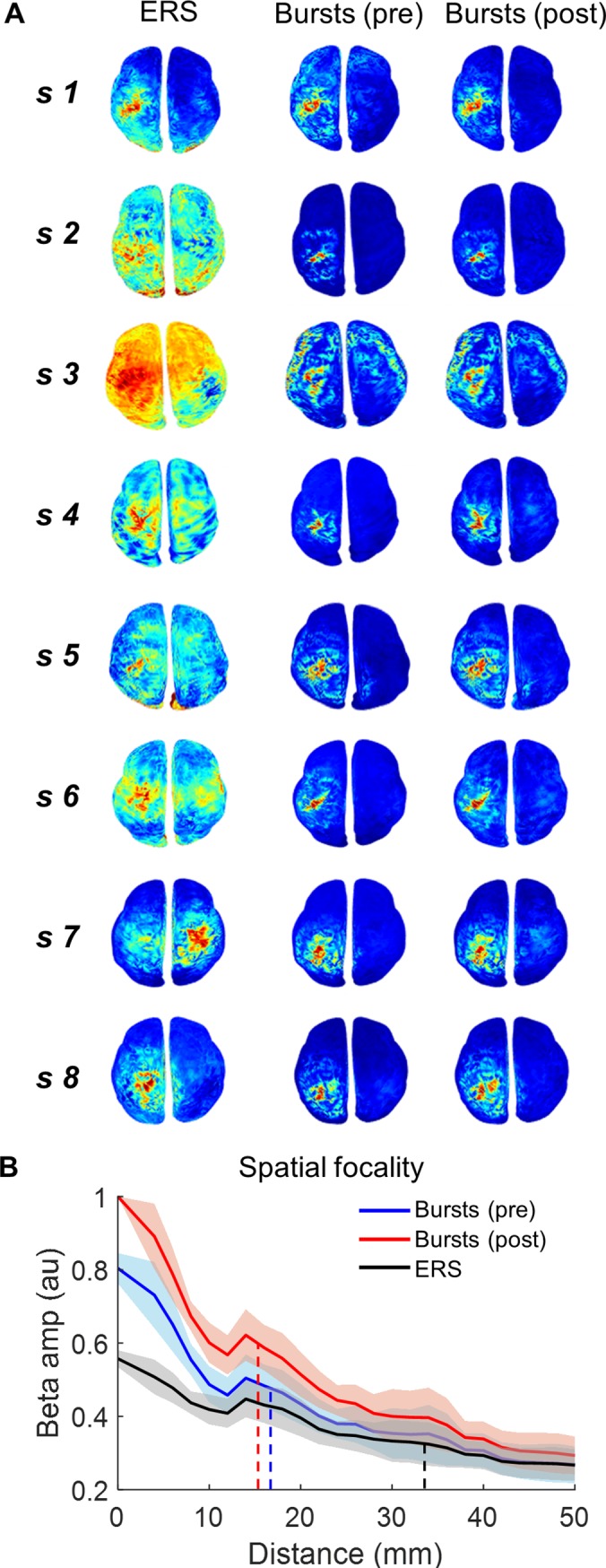
Motor cortical beta bursts are spatially focal. **(A)** Spatial topography of the average beta amplitude in source space for each individual subject (s1–s8), for the subject-specific (660.8 ± 12.1 milliseconds post button press) peak of the ERS, and the average burst for the pre- and post-movement periods, respectively. Colour ranges are individualised to highlight the spatial spread of activity independent of individual differences in SNR and absolute beta amplitude levels. Red colouration represents maximum activity and blue the minimum amplitude per subject (performed independently for ERS (left) and burst analyses (middle and right plots). Data are not baseline normalised within subject. Beta bursts have a more focal topography than the ERS (see also S1 Video and S2 Video). **(B)** Spatial focality of bursts versus average ERS across all subjects. The normalised decrease in beta amplitude with distance from left M1, for all subjects ± SEM (normalised within the subject by dividing the amplitude by the post-movement average burst maxima at the centre of M1), for the ERS, and the pre- and post-movement bursts. This confirms the faster drop-off in beta amplitude with distance from M1 for the bursts (FWHM [dashed line]: pre-movement (blue) −17.5 ± 2.7 mm; post-movement (red) −15.5 ± 2.2 mm), compared with the average ERS (FWHM [black]: 33.5 ± 5.2 mm), indicating increased amplitude and spatial focality. The underlying data can be found at https://osf.io/eu6nx/. ERS, event-related synchronisation; FWHM, full width half maximum; SNR, signal-to-noise ratio.

Having demonstrated that beta bursts are large in amplitude, transient, and account for the majority of the variance in classical trial-averaged beta signals, we proceeded to examine the spatial topography of beta bursts. Average movement-related beta changes pre- and post-movement are generally spatially distributed and often bilaterally observed. We analysed the spatial topography of the ERS movement-related beta change in source space for each subject. Beta amplitude (percent normalised) was projected onto subject-specific brain meshes and colour normalised between the subject-specific ERS maximum (red) and minimum (blue; left panels Fig 6A). During the post-movement (ERS) period (left panels Fig 6A), the average increase in beta amplitude was spatially diffuse and bilateral in the majority of subjects, echoing previous reports, although with notable heterogeneity across subjects (left panels Fig 6A) [13,60].

In order to show a map of an average beta burst, we derived the spatial topography for each burst individually during the peak of the burst amplitude. These spatial topography maps were then averaged separately for the pre- and post-movement bursts and the mean burst spatial topography map also projected onto individualised brain meshes with subject-specific colour ranges, with average burst maximum (red) and average burst minimum (blue). On visual inspection, this appeared to demonstrate a similar topography of bursts between pre- and post-movement epochs, although post-movement bursts contained slightly greater amplitude. We formally quantified this by examining the reduction in normalised amplitude as a function of distance from the primary motor cortex, for the ERS peak and for individual bursts (separately for pre- and post-movement bursts). Note that the peak height of the average burst is greater than the maximum ERS (Fig 6B), and the decrease in amplitude with increasing distance away from the centre of the primary motor cortex was steeper (i.e., more focal) for bursts compared with the mean ERS period beta activity. For the pre- and post-movement bursts, the full width half maximum (FWHM) was, on average, 17.5 ± 2.7 mm and 15.5 ± 2.2 mm, respectively. By contrast, for the conventional trial-averaged peak ERS, the FWHM was significantly greater (more diffuse) at 33.5 ± 5.2 mm (*t*(7) = 7.4, *p* < 0.001 and *t*(7) = 7.8, *p* < 0.001).

Burst topography in the sensor domain (single dipole) as well as analyses of the consistency of bursts across subjects are shown in S3 Fig. To measure the consistency between individual bursts, we cross-correlated each individual burst with the canonical (subject average) burst. The peak cross-correlation (from the centre point ± 25 milliseconds to allow for temporal jitter; see Materials and methods) was then averaged across all bursts. This demonstrated mean (peak) cross-correlation values of 0.39 ± 0.03 and 0.44 ± 0.03 for bursts taken from the pre- and post-movement periods, respectively. The moderate correlation values found here suggest that bursts have a degree of similarity to each other (in keeping with the range of burst lengths shown above [Fig 3B]).

### Beta bursts index motor preparation

Having demonstrated that conventional pre-movement beta ERD and post-movement ERS are dominated by punctate, nonrhythmic, spatially focal, high amplitude burst events, we proceeded to examine the putative functional relevance of these beta burst events.

**Fig 7:**
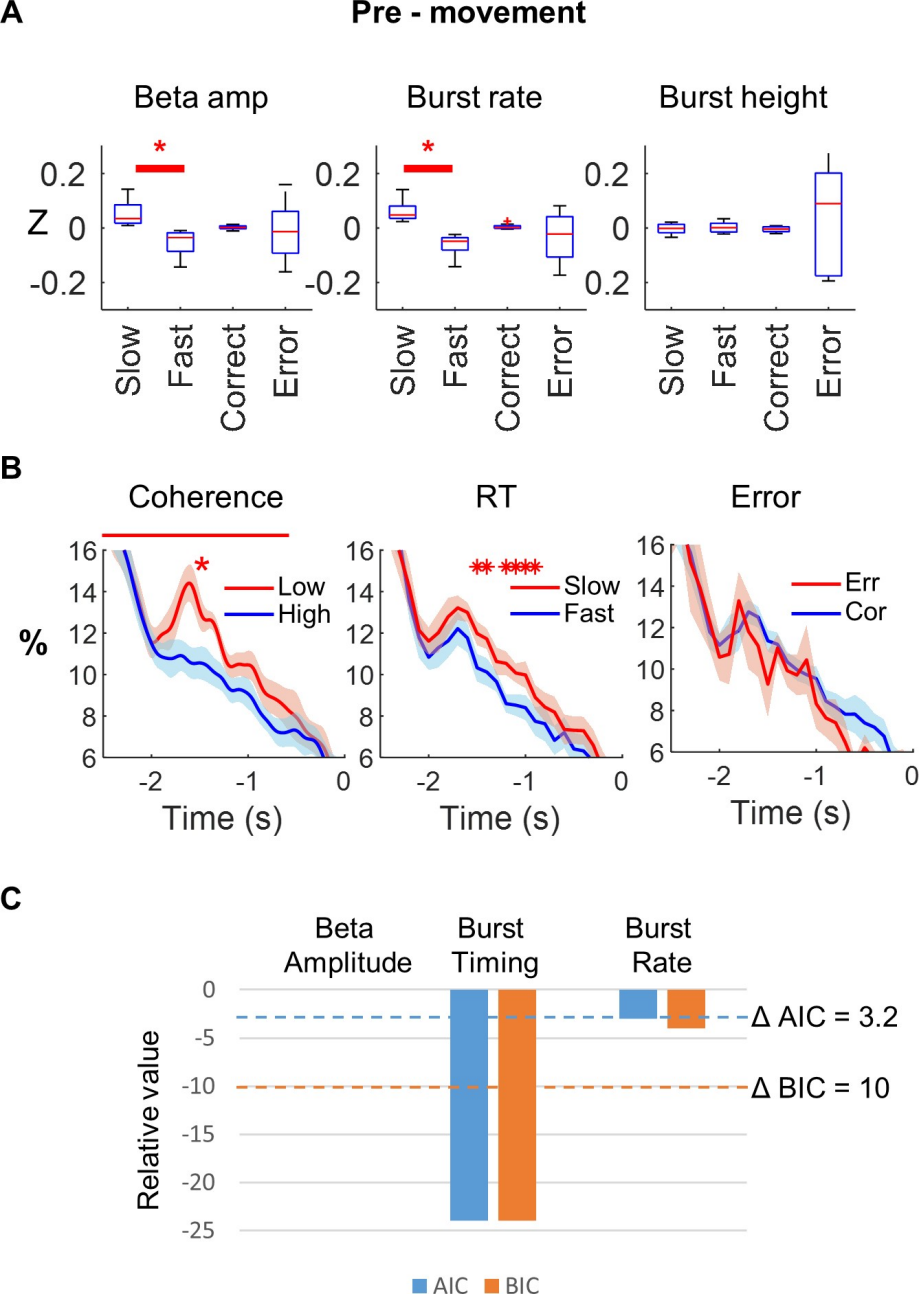
Relationship between beta and motor preparation pre-movement. **(A)** Pre-movement beta amplitude, burst rate, and burst height, z-score normalised within subject and split according to trials with slow versus fast response times and correct versus incorrect trials (*p_FDR_ < 0.05). This shows a higher beta amplitude and burst rate on slow trials pre-movement but no effect of upcoming error. **(B)** Pre-movement burst probability is greater on low coherence than high coherence trials over the RDK period (−2.5 to −0.5 seconds; *t*(7) = 4.3; *p* = 0.004; left panel). Burst probability was also higher in trials with a slow versus fast response time between 1,500 → 900 milliseconds before movement onset (red asterisks, FDR corrected; middle panel), with no effect of upcoming error on burst probability (right panel). On the time axis, zero indicates presentation of the imperative stimulus. Data are down-sampled to 20 Hz. **(C)** Model comparison of three separate linear mixed effects models incorporating one of single trial beta amplitude (1), beta burst timing (2), and burst rate (3), separately, for response time in the pre-movement period. ΔAIC and ΔBIC model comparison metrics are shown (relative AIC and BIC values compared with those of the beta amplitude model). Conventional thresholds for differences are shown for AIC (blue dashed line: −3.22) and BIC (red dashed line: −10), which is equivalent to a 5× difference in probability of the models or a Bayes factor of 150, respectively. The burst timing model shows a significantly lower AIC/BIC compared with the beta amplitude model (ΔAIC = −24) and (ΔBIC = −24) for the pre-movement period, confirming that burst timing is a better predictor of response time than average single trial beta amplitude. The underlying data can be found at https://osf.io/eu6nx/. AIC, Akaike information criterion; BIC, Bayesian information criterion; FDR, false discovery rate; RDK, random dot kinematogram; RT, response time.

We manipulated the degree of preparedness prior to movement through the different levels of coherence in our RDK cue stimuli, and contrasted these levels with burst probability across trials. One-way repeated measures ANOVA revealed a significant effect of RDK coherence on burst probability pre-movement (F(2,14) = 14.4, *p* = 0.0004). Post hoc analysis demonstrated that burst probability was significantly greater in the low RDK versus the high RDK coherence trials (RDK period; *t*(7) = 4.3; *p* = 0.004). Bursts occurred more often during low compared with high RDK coherence (left panel Fig 7B), suggesting a functional relationship between burst occurrence and processes related to motor preparation and initiation. We next proceeded to examine this relationship in more detail.

### Beta bursts are associated with delayed motor initiation and correct responses

**Fig 8:**
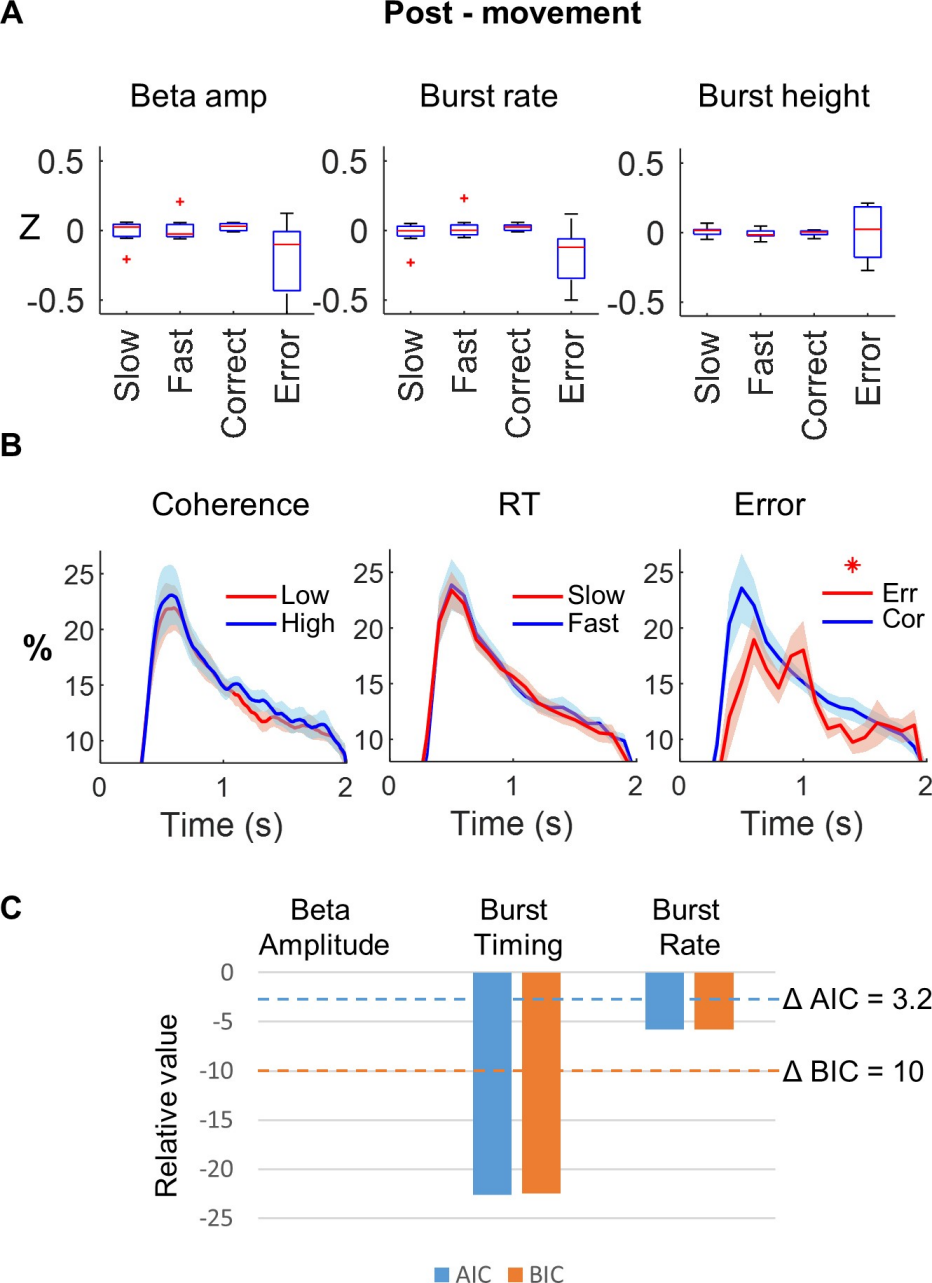
Relationship between beta and errors post-movement. **(A)** Post-movement beta amplitude, burst rate, and burst height, z-score normalised within subject and split according to trials, with slow versus fast response times and correct versus incorrect trials. Note the decrease in beta amplitude and burst rate on error trials, although this was not significant following FDR correction. **(B)** Post-movement, there was no significant difference between beta bursts on low and high coherence trials (left panel) or slow versus fast trials (middle panel). Conversely, burst probability (*p_FDR_ < 0.05) was higher for correct (blue) versus error (red) trials across all subjects. The difference at the first peak (600 milliseconds) was nonsignificant following FDR correction, whereas the second peak at 1.4 second remained significant. On the time axis, zero represents the motor response time. Data are down-sampled to 20 Hz. **(C)** Model comparison of three separate linear mixed effects models incorporating one of single trial beta amplitude (1), beta burst timing (2), and burst rate (3) for response accuracy in the post-movement periods. The burst timing model shows a significantly lower AIC/BIC compared with the beta amplitude model (ΔAIC = −24) and (ΔBIC = −24) for the post-movement period, confirming that burst timing is a better predictor of response accuracy than average single trial beta amplitude. The underlying data can be found at https://osf.io/eu6nx/. AIC, Akaike information criterion; BIC, Bayesian information criterion; FDR, false discovery rate; RT, response time.

Average, across-trial, z-scored beta amplitude, burst rate, and burst height were first examined according to fast versus slow trials and correct versus incorrect trials (Fig 7A and 8A). This shows a higher beta amplitude and burst rate on slow trials pre-movement (beta amplitude *t*(7) = 3.2, *p* = 0.015, *p*_*FDR*_ = 0.02; beta burst rate t(7) = 4.5; *p* = 0.003, *p*_FDR_ = 0.009; Fig 7A). Post-movement beta amplitude and burst rate were lower on error trials, although this was not significant following false discovery rate (FDR) correction for either beta amplitude (*t*(7) = 2.1, *p* = 0.07, *p*_*FDR*_ = 0.1) or beta burst rate (t(7) = 2.7, *p* = 0.03, *p*_*FDR*_ = 0.09; Fig 8A). However, even if average beta (within or across trials) might represent a reasonable summary metric of average burst occurrence, this will disguise the precise timing of beta bursts on a trial-by-trial basis, which would be expected to be behaviourally relevant if bursts contribute to direct communication between brain regions [61–63] or local cortical circuit processing [58].

Our further hypothesis is, therefore, that beta burst timing relates to behaviour. If this is indeed the case, then pre-movement burst timing on individual trials should relate to RT, whereas beta burst timing after movement might be associated with the commission of errors. Moreover, such a relationship would be concealed in conventional, trial-averaged accounts of beta amplitude, suggesting that burst timing should provide a better explanation of behaviour. We therefore examined the time course of burst occurrence by comparing the burst probability (over trials) of the fast versus slow response time trials across all subjects at each time point during the presentation of the predictive RDK stimulus. This indexes the time during the motor preparation period when the occurrence of a burst affects future movement initiation. We found that within a window between −1,500 milliseconds and −900 milliseconds (relative to the imperative stimulus), the occurrence of bursts during this period predicted slower subsequent response times (*p* < 0.05, FDR corrected, Fig 7B). In contrast, pre-movement burst occurrence was not related to movement errors (*p* > 0.05, Fig 7B) at any time during motor preparation.

In the post-movement period, we contrasted the burst probability at each time point for correct versus incorrect trials. We observed two periods of lower probability of burst occurrence for incorrect trials at 600 milliseconds (*p* = 0.026; *p*_*FDR*_ = 0.19) and at 1,400 milliseconds (*p* = 0.0022; *p*_*FDR*_ = 0.046, Fig 8B), the latter of which survived FDR correction. Post-movement burst occurrence did not correlate with previous response time (*p* > 0.05, Fig 8B).

### Beta burst timing is a stronger predictor of behaviour than burst rate or single trial beta amplitude

We next sought to directly compare burst timing, burst rate, and single trial beta amplitude changes in a model together in order to determine the strongest predictor of behaviour. We hypothesised that the timing of bursts that were temporally closely aligned to movement might have the strongest impact on behaviour. Therefore, on each trial, we determined the timing of the last burst prior to the imperative stimulus (movement preparation) and the timing of the first burst after the button press (movement execution).

Across all subjects, the timing of the last burst prior to the imperative stimulus occurred at −936.7 ± 90 milliseconds. However, within individual subjects, the timing of this last burst was widely dispersed, with a mean within-subject standard deviation (across all trials for that subject) of 673.0 ± 36.6 milliseconds. Bursts also occurred earlier overall on fast compared with slow response trials in 6 out of 8 subjects (mean: 42 ± 22 milliseconds).

To determine the effects of single trial burst timing, rate, and beta amplitude on response time, we used a linear mixed model with z-scored burst rate, burst time, and burst amplitude, as well as stimulus coherence and congruence and their interaction as fixed effects, and subject-specific offsets as random effects. The dependent variable was the (log-transformed) response time. Mixed models have the advantage of utilising all of the trial data rather than averaging over trials within each condition, and assess the relationship between fixed effects and the dependent variable at the single trial level. This pre-movement analysis showed a significant effect of burst timing (F(1,11558) = 28.90, *p* < 0.001) and amplitude (F(1,10973) = 11.69, *p* < 0.001), but not burst rate (F(1,11561) = 1.37, *p* = 0.241). Variance inflation factors for all fixed effects were less than 2.0, indicating that multicollinearity was not too severe for inclusion of all variables in the model (beta amplitude = 1.21, burst rate = 1.48, burst timing = 1.25).

In the post-movement period, the first burst occurred 683.6 ± 34.1 milliseconds after the button press across all subjects. However, again we found that within individual subjects and trials, the timing of the first burst was widely dispersed, with a mean within-subject standard deviation of 405.0 ± 12.4 milliseconds. The timing of the first post-movement beta burst occurred later on error trials compared with correct trials in all 8 subjects (mean: 105 ± 17 milliseconds). We used a generalised linear mixed effects model to assess the combined effect of trial-wise beta amplitude, burst rate, and the timing of the first burst following button press on error commission (0 → 2,000 milliseconds). Post-movement, only the burst timing (χ^2^(1) = 20.72, *p* < 0.001) and single trial beta amplitude (χ^2^(1) = 4.19, *p* = 0.041) showed significant relationships to error, while single trial burst rate did not (χ^2^(1) = 1.05, *p* = 0.305). Variance inflation factors for all fixed effects were again less than 2.0 (beta amplitude = 1 .177, burst rate = 1.44, burst timing = 1.27).

In order to determine the relative predictive relationships of the three variables, we also analysed these data for each individual predictor in a separate model and then performed model comparison. In the three additional models, we analysed either burst timing, single trial burst rate, or single trial beta amplitude separately, including stimulus coherence and congruence and their interaction as additional fixed effects. As these models are non-nested, we compared them using the Akaike information criterion (AIC) [64,65] and Bayesian information criterion (BIC) metrics [66]. Pre-movement, the relative differences in AIC and BIC compared with the single trial beta amplitude model were ΔAIC of −24, and ΔBIC of −24, for the burst timing model, and ΔAIC of −3, and ΔBIC of −4, for the burst rate model (Fig 7C). Post-movement, the relative differences in AIC and BIC (compared with the single trial beta amplitude model) were ΔAIC of −22.6, and ΔBIC of −22.5, for the burst timing model, and ΔAIC of −5.8, and ΔBIC of −5.8, for the burst rate model (Fig 8C). A lower AIC or BIC is evidence for a better model, and a difference of AIC < −3.22 indicates that one model is five times more likely than the comparator model, whereas a BIC difference of −10 is equivalent to a Bayes factor of 150. Model comparison therefore revealed a stronger link between beta burst timing and behaviour, compared with beta burst rate or single trial beta amplitude.

At the single trial level, the timing of beta bursts prior to movement thus predicts the response time, whereas post-movement, it indexes the commission of errors. When considered alone, beta amplitude across the entire pre- and post-movement periods also predicts response time and indexes response errors. However, the predictive power of the timing of the last burst prior to the imperative cue and of the first post-movement burst was greater than that of single trial beta amplitude. Beta bursts and their timing, rather than average (both within and across trials) beta amplitude per se, might therefore be key to understanding the functional relevance of beta and its relationship to the motor system.

## Discussion

Movement-related activity changes in the beta frequency band (13–30 Hz) are a hallmark feature of healthy and pathological movement, yet their functional role remains debated [9,58,67,68]. Overcoming this knowledge gap is critical to understanding the physiological underpinnings of healthy and pathological movement, and to inform the development of new treatments for diseases of movement that show pathophysiological beta activity [25,69,70].

### Analyses of average beta amplitude conceal burst timing dynamics

It is increasingly becoming apparent that average beta changes in the motor system may be a simplistic and static summary of the underlying neural dynamics [42,45,57,71]. Further understanding of the role of these signals mandates detailed decomposition of the average changes of rhythmic activity into the underlying dynamics at a single trial level and implementation of appropriate methods to capture rapid bursting activity. By leveraging high-precision MEG, we here show that motor cortical beta activity in healthy participants at the single trial level is highly dynamic and dominated by bursts of short-lasting (100–200-millisecond) beta activity, with limited evidence of sustained, amplitude-modulated oscillations. Crucially, even though beta bursts encompass a relatively small fraction of the total time in a given trial (pre-movement, 11.9%; post-movement, 12.7%), their timing provides a better explanation of behaviour in the present task than the beta amplitude, even at the single trial level.

### Beta burst timing before and after movement

In the pre-movement period, we found that less informative preparatory cues are associated with a higher probability of beta bursts. Notably, the presence of bursts and late timing of these bursts prior to movement predict slower movement initiation. By contrast, in the post-movement period, the commission of errors results in delayed onset of bursts, and a reduced probability of beta bursting. In both the pre- and post-movement periods, beta burst timing showed a stronger relationship to behaviour than beta amplitude changes, even at the single trial level. Importantly, this timing relationship with behaviour is concealed in conventional analyses of averaged beta amplitude. The short-lasting beta burst dynamics pre- and post-movement suggest a functional role that goes beyond merely supporting a sustained process, such as pre-movement inhibition or prolonged generalised states of movement preparation. Such a process would be compatible with sustained (slow) and spatially diffuse beta changes. As we show here, the dominant signals at a single trial level are temporally sparse and spatially focal bursts, whose rate and timing predict motor responses and index errors.

Our results suggest a link between motor cortical beta burst activity and processes related to movement preparation and planning [17,42,47,72], as well as post-movement updating processes that track sensory cues and movement outcomes [10,15,73]. This extends recent work on the short-lasting temporal dynamics of beta in the human sensory cortex, basal ganglia, and nonhuman motor system [43,45,47,54,74] and their mechanistic relevance in the motor system [35–37,75,76]. However, our results motivate a reappraisal of the divergent classes of interpretations of beta activity, which have often relied on trial-averaged beta analyses [1,32,40,77,78]. Rather than beta activity subserving a sustained and generalised inhibitory role, bursts may have a specific information-encoding role that can rapidly bias behaviour, even though bursts are short lasting and not necessarily strictly time-locked to the response or visual cue. Such a view sides with recent proposals that link beta activity to processes such as response selection [10,12,79] or top-down hierarchical cortical processing [63,80–82].

### A role for beta bursts in movement goal selection and updating?

High amplitude beta bursts at the cellular and network levels signify synchronous recruitment of neuronal populations that fire together over just a few cycles [83]. Furthermore, a dominant theory in cognitive neuroscience is that synchronised neuronal activity may be critical for communication [62,63]. However, our findings raise the question: how do such transient neocortical beta events contribute to information processing? Previous work has proposed that local, transient beta signals reflect brief synaptic inputs to local cortical layers, which may influence behaviour via a decrease in efficacy of top-down information expressed via supragranular pathways [47] or a decrease in the influence of bottom-up information flow via the thalamus [47,48,58]. If the timed synaptic events underpinning beta bursts do indeed control the balance between local and incoming information, it will be critical to determine in more detail how this might contribute to the control of movement. Speculatively, if seen as a communication and integrating mechanism, beta bursts may not reflect the specific local computations necessary for movement per se, but represent brief periods of interregional communication [46], which could include functions such as goal formation or post-performance evaluation and integration [10,15,73]. Such a view would be broadly compatible with the idea that strongly correlated neural spiking, as is the case for beta power increases, reduces the capacity for local information processing [84] but could open up channels for longer range communication [85]. Recent evidence suggests that during periods of beta bursting, rapid interregional phase locking occurs, starting just prior to and for the duration of the burst, providing a direct possible mechanism of how such interregional communication could occur [71]. Our work, however, does not directly resolve the specific circuit mechanisms through which beta bursts contribute to information processing. Fine-grained neural network models, together with direct recordings and pharmacological or stimulation manipulations, will help to arbitrate between competing accounts in future work [73].

Subcortical beta bursting is now a target for developing adaptive deep brain stimulation (DBS) technologies that seek to directly reshape the temporal profile of beta events and thereby provide evidence for their causal role [70,74,86–88]. If effective beta burst–triggered adaptive DBS could be demonstrated at the cortical level, this could validate the causal, mechanistic effects of beta bursts on behaviour. In addition, it would also open up treatment possibilities for neurological disorders characterised by aberrant cortical beta activity [27,89]. Interestingly, emerging evidence suggests that other frequency bands may also be better characterised by bursting rather than sustained oscillations (S4 Fig), in particular, gamma and theta frequency activity [44,90,91]. Whether short-lasting oscillatory bursts over the full range of frequencies represent a widespread mechanism of endogenous, local, content reactivation [58] or can also support long-range communication [61,63], more generally remains to be determined. Here, we specifically focused our analysis on beta burst rate and timing in human motor cortex, and demonstrate that these can account for the changes seen in the ERD and ERS and their functional impact on the control of movement, even at the single trial level.

### Conclusion

Single trial beta activity in motor cortex is dominated by brief, high amplitude, sporadic bursts of activity. These bursts are spatially more focal than average beta changes and are functionally coupled to movement. Pre-movement beta bursts relate to the degree of preparation of a forthcoming action, based on visual cues, and are associated with delayed movement initiation. By contrast, post-movement bursts are linked to performance, being delayed on error trials. These results suggest a reappraisal of classical interpretations of beta activity to incorporate the transient, temporally distributed nature of beta bursts.

## Materials and methods

### Ethics statement

Eight healthy subjects participated, following informed written consent, which was approved by the UCL Research Ethics Committee (reference number 5833/001). This study was conducted in accordance with the Declaration of Helsinki.

### Visually cued movement selection task

All subjects were right-handed, had normal or corrected-to-normal vision, and had no history of neurological or psychiatric disease (6 male, aged 28.5 ± 8.52 years). Participants performed a visually cued movement selection task, with a preparation phase followed by an imperative stimulus. Movements consisted of a left or right button press, all made with the index and middle fingers of the right hand. As we aimed to study short-lasting beta bursts during movement selection, we elected for a preparation cue that was sustained (RDK), so as to minimise the production of neural transients. Therefore, after a baseline period of 1–2 seconds, during which participants fixated on a small white cross, an RDK was displayed for 2 seconds, with coherent motion either to the left or the right indicating the likely upcoming required movement. The RDK consisted of a 10˚ x 10˚ square aperture centred on the fixation point, with one hundred 0.3˚-diameter dots, each moving at 4˚/s. The RDK preparation cue predicted the direction of the upcoming imperative stimulus on 70% of trials, thus allowing participants to prepare (but not initiate) their response in advance of the subsequent imperative stimulus. On any given trial, a certain percentage of dots (motion coherence level) moved through the aperture in one direction, left or right, with the remainder of the dots moving randomly but on a consistent path. The motion coherence level was set individually for each subject using an adaptive staircase procedure to find a level at which they achieved 82% accuracy in a block of 40 trials at the beginning of each session (QUEST; [92]). In order that we could control the difficulty of movement preparation and analyse the effect of this on bursts, we used three levels of coherence—a medium coherence as defined by the procedure above, then a low coherence (50% of medium) and a high coherence (150% of medium). Each block consisted of 180 trials, with 60 trials at each coherence level and 126 and 54 congruent and incongruent trials, respectively. The paradigm was implemented using the Cogent 2000 toolbox through MATLAB (The MathWorks, Natick, MA, http://www.vislab.ucl.ac.uk/cogent.php).

### Magnetic resonance imaging and head-cast fabrication

Subjects underwent both a standard MRI scan for individualised head-cast creation and a high-resolution, quantitative, multiparameter map scan [93], which was used for accurate cortical surface extraction and source reconstruction. In order to record beta activity with a maximal level of spatial precision, we used subject-specific head-casts, which have been shown to optimise co-registration, reduce head movements, and thereby significantly improve SNR (see S1 Text) [49,50].

### MEG scanning

Subjects underwent MEG recordings (CTF 275 Omega system) using their individual head-casts during a visually cued, movement selection task. Head position was localised using three fiducial coils placed at the nasion and left/right pre-auricular points, within the head-cast. Data were sampled at 1,200 Hz before being imported to SPM 12 and subsequently down-sampled to 250 Hz.

### Source inversion

For source analysis, sensor-level data were inverted onto subjects’ individual cortical surface meshes. These meshes were extracted using Freesurfer [94] from multiparameter maps using the PD and T1 sequences as inputs, with custom modifications of the reconstruction to avoid tissue boundary segmentation failures [95]. Each mesh was then down-sampled by a factor of 10 (approximately 33,000 vertices per mesh) and smoothed (5 mm).

Source reconstruction (estimation of current dipole positions and strengths) was performed using SPM12 and an Empirical Bayesian beamformer (with an additional analysis for comparison by minimum norm inversion; S1 Fig) without Hann windowing, using a single shell model and a frequency of interest of 1–90 Hz [96].

### Time frequency decomposition

In order to obtain the beta amplitude trace, the time series virtual electrode data (see S1 Text) were filtered using a fourth-order (two pass) Butterworth filter with a frequency range of 13–30 Hz. A complex (analytic) function was then extracted from the time series using the Hilbert transform and the amplitude derived by taking the modulus of that signal.

### Comparison of beta amplitude dynamics to surrogate data

We first analysed the variability in beta amplitude (envelope) separately for the periods prior to and after movement, in order to determine if this was more or less dynamic than would be expected using a spectrally matched surrogate dataset. To this end, we quantified the CV (CV = std beta amplitude / mean beta amplitude) on each trial separately for the pre- and post-movement periods, respectively. We tested whether there was a significant difference in CV between real data and surrogates using paired *t* testing. For visualisation, we plotted a histogram of average CV values for all subjects (Fig 3A). Our surrogate data were a spectrally matched, phase-randomised dataset that retained all of the spectral features of the original data but randomised the starting phase of the individual sinusoids (in the frequency domain) and therefore redistributed the (same) beta amplitude across time. This procedure produces a null distribution of many different possible time series that can result in the same power spectrum (function: spm_phase_shuffle; SPM12) [42].

### Analysis of beta rhythmicity

We measured rhythmicity using lagged coherence [59], which is a frequency-specific measure of the coherence between a signal and a lagged version of the same signal. This involves segmenting the signal into non-overlapping epochs with frequency-dependent widths. Fourier coefficients are then obtained for each epoch using a three-cycle Hann-windowed Fourier transform. The phase relations between pairs of epochs are then then computed, either between adjacent epochs or epochs separated by a longer lag. The sum of these phase relations over all epoch pairs is then normalised to yield the lagged coherence, which is 0 when there is no phase consistency between epochs and 1 when there is perfect phase consistency. For comparison with lagged coherence, we computed power spectra using Welch method PSD estimation. A 1/f function was then fit to the resulting PSDs, and the standardised residuals (raw residuals divided by their estimated standard deviation) were plotted to show oscillatory power above aperiodic background noise. The lagged coherence and power analyses used a cycle-shuffled surrogate dataset as a control, which preserved the power spectrum of the original signal, but randomly shuffles peak-trough data segments of the time series to remove correlations between cycle phase [97,98].

### Empirically defining beta burst thresholds

Thresholds for identifying beta bursts were defined, for each participant, by correlating the trial-wise beta amplitude with the probability of beta burst occurrence over time within an epoch (beta burst count), across a range of different threshold values [45,55,88,99]. The various thresholds ranged from the median beta amplitude to median amplitude plus five times the standard deviation of the beta amplitude (see S1 Text for detailed description). This analysis was repeated separately for the pre- and post-movement periods, respectively. In our data, the maximum (peak) of the average correlation curve was found to be 1.75 standard deviations above the median beta amplitude for both the pre-movement and post-movement periods. The 1.75-SDs threshold was then used for each subject, so that their relative thresholds were matched at 1.75 SDs above the subject-specific median of the beta amplitude (noting that absolute thresholds could differ across subjects according to their data and SNR).

### Beta burst rate and timing

A priori, we hypothesised that the occurrence of bursts and their timing would both influence behaviour.

Burst rate indexes the number of bursts within a given period (e.g., 2,500 milliseconds prior to the imperative stimulus) on any given trial. This was calculated here, as previously it has been suggested that within-trial burst rate might itself be a coding scheme for tactile sensation [45,47].

In addition, burst probability (at each time point across trials) was calculated by binary coding all trial time points as to whether the beta envelope was either above or below threshold on each individual trial. This was averaged over trials and scaled by the total trial number to give a time-resolved probability of bursts per subject. Time-resolved ERD and ERS were calculated by averaging beta amplitude across trials, and this average value was then normalised as a percent change to baseline in order to facilitate comparison across all subjects (Fig 5). The baseline was the same for both the pre-movement and post-movement analyses and was the 500 milliseconds prior to presentation of the preparation (RDK) cue (during presentation of the fixation cross).

The ERD and ERS (over time) was then correlated against the burst probability curve (over time) for each subject to derive an *R*^2^ value of correlation between the two. This is a measure of how the shape of the time-resolved burst probability (black trace Fig 5) matches that of the ERD and ERS (red trace Fig 5) during the pre- and post-movement periods, respectively.

### Quantification of beta burst amplitude and length

Bursts were defined by periods at which the beta amplitude exceeded our empirically defined threshold (median + (1.75 × SD)). The height of individual bursts was defined by the maximum of the peak amplitude during the period above threshold. Burst duration was defined as the time (milliseconds) over which the amplitude remained above threshold. These features were then compared with surrogate data using the phase-randomised dataset (as described above) and the same threshold. If beta bursts are a significant neural coding feature, we would expect there to be a greater number of larger, more sustained bursts in the real data as compared with surrogate data when the time series is phase-randomised.

The differences in the burst characteristics between the two different datasets (same threshold used for burst definition in both datasets) were plotted for each subject on a 3D histogram (Fig 3B). We then tested the hypothesis that beta bursts were larger than would be expected by comparing the distributions of burst heights and burst lengths for 10 different heights (percentiles) and lengths (100-millisecond bins up to 1,000 milliseconds) against our surrogate, spectrally matched distributions (mean of ×1,000 iterations) using paired *t* tests across subjects (Fig 3B; FDR corrected).

### Analysis of the spatial distribution of beta bursts in motor cortex

We first examined the spatial distribution of the average beta amplitude across trials and plotted this for each subject for the end of ERD and then also around the subject-specific ERS. This was then compared with the spatial topography of the burst amplitude at their peaks, which was calculated for each burst individually and then averaged over all bursts. The ERD epoch was defined as the final 200 milliseconds prior to button press, and the ERS epoch was defined as 100 milliseconds either side of the subject-specific average maximum post-movement beta (i.e., the classical ERS).

All plots show non-normalised beta amplitude to avoid distortion but with subject-specific colour bars. The colour bars were set individually for each subject in order to best highlight relative spatial topography. The range was taken from the beta amplitude values at all vertices in the whole brain above the midline (in order to avoid distortion from deep artefacts that can occur during source inversion), with red indicating the maximum amplitude and blue indicating the minimum amplitude for that subject.

Having visualised the spatial topography of beta for each subject, we next wanted to quantify the drop-off in beta amplitude with distance away from the M1 hand area, to compare this between the classical trial-averaged beta amplitude and the average beta burst for each subject.

Therefore, we sorted each vertex according to its Euclidean distance from the central M1 vertex and binned them in 5-mm bins (up to 50 mm). The amplitude at these vertices (without baseline normalisation) was then averaged according to distance (within each 5-mm bin). This was performed first on the average amplitude across trials during the period surrounding the subject-specific peak amplitude post-movement (±100 milliseconds; i.e., the ERS). Secondly, this was repeated for each burst at its amplitude peak and averaged over pre- and post-movement bursts separately. The FWHM was then calculated within individual subjects for the ERS and for bursts as the distance away from the centre of the M1 hand area, for which the amplitude had dropped by half. The FWHM value was compared between the classical ERS and the pre- and post-movement bursts by *t* testing. For cross-subject visualisation, we normalised these data for each subject by dividing all values by the amplitude of the first bin (0–5 mm).

### Beta burst shape and consistency

To analyse the shape and consistency of the bursts, we realigned the data to the centre of the peak of the amplitude. Unfiltered source-level data were re-epoched around the amplitude peaks with a window of 100 milliseconds on either side (S3 Fig). This burst aligned (unfiltered) dataset was then averaged across all bursts to look for consistency of the waveform (in the time domain) within the amplitude aligned bursts, and this was repeated with surrogate data (S3 Fig). Individual bursts were then cross-correlated against the average burst and the maximum r value (max(r)) within 25 milliseconds (half a cycle) before or after the midpoint (to allow for some a small amount of jitter = approximately 1 beta cycle), taken as a measure of the concordance of the individual burst with the average, repeated for the pre-movement and post-movement bursts. The average waveform for each subject was then normalised by the maximum (absolute) value for that subject, and these normalised subject-specific bursts averaged across all subjects, with the time series ± orientation displayed with the minimum closest to time zero, as per previous studies [45,47]. All statistical analyses were performed on the beta amplitude envelope and were therefore invariant to inherent uncertainty in dipole orientation caused by source inversion uncertainty and sulcal variation across subjects. Analyses were repeated in the theta and gamma ranges as functional controls and to check that the behavioural relationships found with beta bursts were specific (S4 Fig).

Finally, the sensor-level field map was examined by averaging the (subject mean normalised) magnetic field for all bursts and subjects for a representative sensor overlying the left motor area (MLT14) at the three moments of the average maximum dipole in the amplitude-aligned dataset. This was then plotted as an average scalp map, which confirmed the presence of a single dipole (spm_eeg_plotScalpData) that was similar for pre- and post-movement periods.

### Behavioural analyses

To analyse the differential contribution of the beta amplitude and the burst characteristics to behaviour, pre- and post-movement periods were analysed separately. Responses that matched the direction of the imperative stimulus were classed as correct, and the response time (RT) was taken as the time difference between imperative stimulus and the button press.

Beta amplitude, burst rate, mean burst height, and mean burst duration were calculated in the pre-movement period (cue locked dataset; start of RDK −2,500 milliseconds → 0 milliseconds imperative stimulus) and post-movement period (button press locked dataset; 0 milliseconds → 2,000 milliseconds) and z-scored across all trials. Z-scored trials were then split according to response time (slow versus fast) and decision error (correct versus incorrect) and compared by paired *t* tests across subjects, separately for both pre- and post-movement datasets, respectively (Figs 7A and 8A). Burst timing was analysed in a similar fashion, taking the timing of the last pre–imperative-cue burst and z-scoring and splitting these values according to slow/fast response time and *t* testing. Similarly, for the post-movement period, the timings of the first burst after button press were z-scored, split according to whether a correct response or error had been made, and *t* tested.

Additionally, in order to examine the critical period of this timing response, the time-resolved burst probabilities across all trials were first down-sampled to a sampling frequency of 20 Hz and then grouped according to behavioural output (response time and error). Pre-movement, the difference between burst probabilities in the fast versus slow trials was then compared by paired *t* testing across subjects for each time point (sampling rate = 20 Hz) and corrected for multiple comparisons using the FDR procedure. Post-movement, the burst probabilities were compared between correct and incorrect trials by paired *t* testing (with FDR correction).

In order to examine the effect of beta amplitude, burst rate, and burst timing on an individual trial-by-trial basis and directly compare their impact on behaviour, we proceeded to take these features forward into linear mixed effects models (R statistics, version 3.4.3). Firstly, for RT (dependent variable, log transformed) we used a linear mixed model with z-transformed beta amplitude, beta burst rate, and beta burst timing, as well as stimulus coherence and congruence and their interaction as fixed effects and a subject-specific intercept as a random effect. Fixed effects for this model were tested using type III Wald F tests with Kenward-Roger approximated degrees of freedom [100]. For the post-movement analysis, we used a generalised linear mixed model with a binomial distribution and logit link function, using correct (true or false) in each trial as the dependent variable, z-transformed beta amplitude, burst rate, and burst timing, as well as stimulus coherence and congruence and their interaction as fixed effects, and subject-specific intercepts as a random effect. Fixed effects for this model were tested using type III Wald χ^2^ tests [91].

## Supporting information

S1 Table Response times and behavioural accuracy for congruent and incongruent trials under all conditions of coherence. C, Congruent; I, Incongruent.
(XLSX)

S1 Fig Analysis of empirical burst thresholds. Related to methods—source inversion and identification of beta bursts through empirically defined thresholds. **(A)** Mean correlation curves ± SEM, for the pre-movement (ERD, blue) period and the post-movement (ERS, red) for beta amplitude versus beta burst rate. **(B)** The analysis is repeated for correlation between the beta power (amplitude squared) and burst rate, which leads to a higher threshold determination and a flatter correlation peak. **(C)** Empirical determination of bursts using Minimum Norm (MMN) source inversion, as opposed to an Empirical Bayesian Beamformer (EBB), suggests a similar threshold approximately 1.75 SDs > median when correlating burst rate versus amplitude. The underlying data can be found at https://osf.io/eu6nx/. ERD, event-related desynchronisation; ERS, event-related synchronisation.
(TIF)

S2 Fig Pre-movement beta burst characteristics versus phase-randomised surrogate data. Group-level line histogram of the log10 mean burst counts for different heights (top left) and lengths (bottom left) for real versus surrogate pre-movement data (*p < 0.05, FDR corrected). Difference in burst height and length counts shown for each subject compared with bursts from a surrogate phase-randomised dataset (burst height/length real data minus burst height/length surrogate data; right panels). This demonstrates a relative reduction in the number of the smallest bursts in the real compared with surrogate pre-movement data, with an increase in larger bursts (same analysis shown for post-movement data in Fig 3). The underlying data can be found at https://osf.io/eu6nx/. FDR, false discovery rate.
(TIF)

S3 Fig Beta bursts pre- and post-movement in sensor space. **(A)** Individual (mean and normalised) bursts from all subjects shown in the time domain, source space (left M1). **(B)** Mean beta bursts (normalised to average ERS burst maximum) across all subjects for real (blue) versus phase-randomised, surrogate (×1,000 randomisations) data (red). **(C)** Sensor-level scalp maps (normalised within subjects and averaged over all bursts/subjects) aligned to three periods of maximum extrema (indicated by vertical dashed lines). These show a single dipole at each extrema. Left panel: pre-movement. Right panel: post-movement. The underlying data can be found at https://osf.io/eu6nx/. ERS, event-related synchronisation.
(TIF)

S4 Fig Burst distribution and behavioural results using theta and gamma bands. **(A)** Raster plots for burst distribution according to button press (vertical blue line) for theta (3–7 Hz; left) and gamma (60–90 Hz; right) for all subjects and all trials (individual subjects divided by red dashed line). **(B)** Amplitude in theta (left) and gamma (right) ranges shown in red, with the probability of bursts in the respective frequency ranges shown in black. The strong burst at button press (0 milliseconds) for theta will include a component of low-frequency movement-related evoked response. There is also a small burst of gamma at movement onset. There was no significant difference found in burst probability according to response time (pre-movement) or decision error (post-movement) for theta or gamma (*p* > 0.05). **(C)** Individual average burst shapes in the time domain (centred on the amplitude peak) show different shapes of activity from beta specific bursts. The underlying data can be found at https://osf.io/eu6nx/.
(TIF)

S1 Video Time-resolved change in average ERD and ERS for a single subject in response-locked data. Time varying average amplitude changes for subject 2. Colour bars are matched to the individualised range for subject 2 (Fig 6) between minimum and maximum beta amplitude at the subject-specific peak ERS. Here, it demonstrates a slow and spatially diffuse reduction in beta during the ERD period and a gradual, bilateral, and sustained increase in activity during the ERS period. Averaging over trials gives the impression of spatially diffuse and sustained beta activity. The underlying data can be found at https://osf.io/eu6nx/. ERD, event-related desynchronisation; ERS, event-related synchronisation.
(MP4)

S2 Video Time-resolved average bursts for a single subject. This shows the average beta burst at the same timescale (subject 2) as S1 Video. Colour bars are matched to the individualised range for subject 2 (Fig 6) between minimum and maximum beta amplitude at the subject-specific peak of the average burst. This indicates a much more rapid and spatially focal burst of activity. Note that this demonstrated average beta burst may not perfectly illustrate individual bursts at the individual trial level either, which will be more heterogeneous and may involve travelling waves [101]. However, this demonstrates that, using a matched analysis, bursts are significantly more rapid than conventional average analysis and, on average, are more focal. The underlying data can be found at https://osf.io/eu6nx/.
(MP4)

S1 Text (DOCX)

## Abbreviations

AIC: Akaike information criterion
BIC: Bayesian information criterion
CV: coefficient of variation
DBS: deep brain stimulation
ERD: event-related desynchronization
ERS: event-related synchronisation
FDR: false discovery rate
FWHM: full width half maximum
MEG: magnetoencephalography
RDK: random dot kinematogram
PSD: power spectral density
SNR: signal-to-noise ratio
