# Supplementary figures and images for: Human motor cortical beta bursts relate to movement planning and response errors
Source: PLoS Biol. 2019 Oct 4;17(10):e3000479. doi: 10.1371/journal.pbio.3000479 (PMC6795457; doi:10.1371/journal.pbio.3000479)

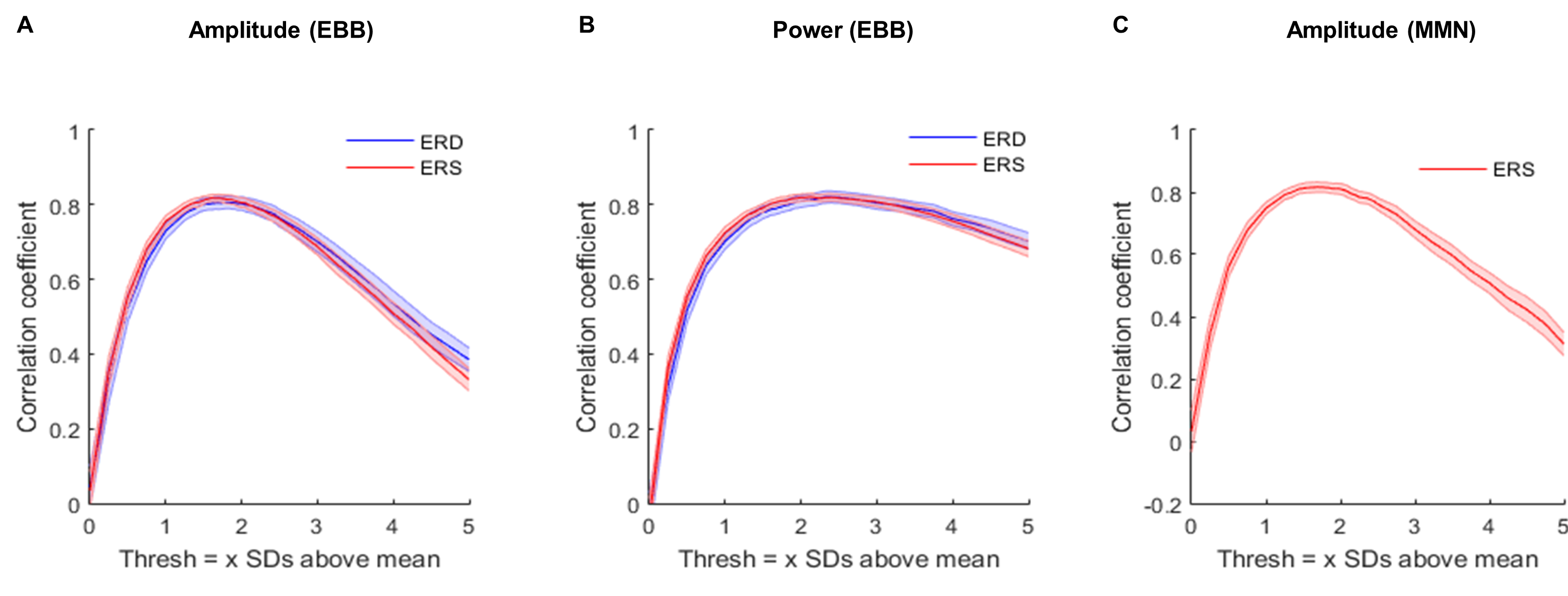

Supplement: S1 Fig — Related to methods—source inversion and identification of beta bursts through empirically defined thresholds. (A) Mean correlation curves ± SEM, for the pre-movement (ERD, blue) period and the post-movement (ERS, red) for beta amplitude versus beta burst rate. (B) The analysis is repeated for correlation between the beta power (amplitude squared) and burst rate, which leads to a higher threshold determination and a flatter correlation peak. (C) Empirical determination of bursts using Minimum Norm (MMN) source inversion, as opposed to an Empirical Bayesian Beamformer (EBB), suggests a similar threshold approximately 1.75 SDs > median when correlating burst rate versus amplitude. The underlying data can be found at https://osf.io/eu6nx/. ERD, event-related desynchronisation; ERS, event-related synchronisation. (TIF) [file pbio.3000479.s002.tif]

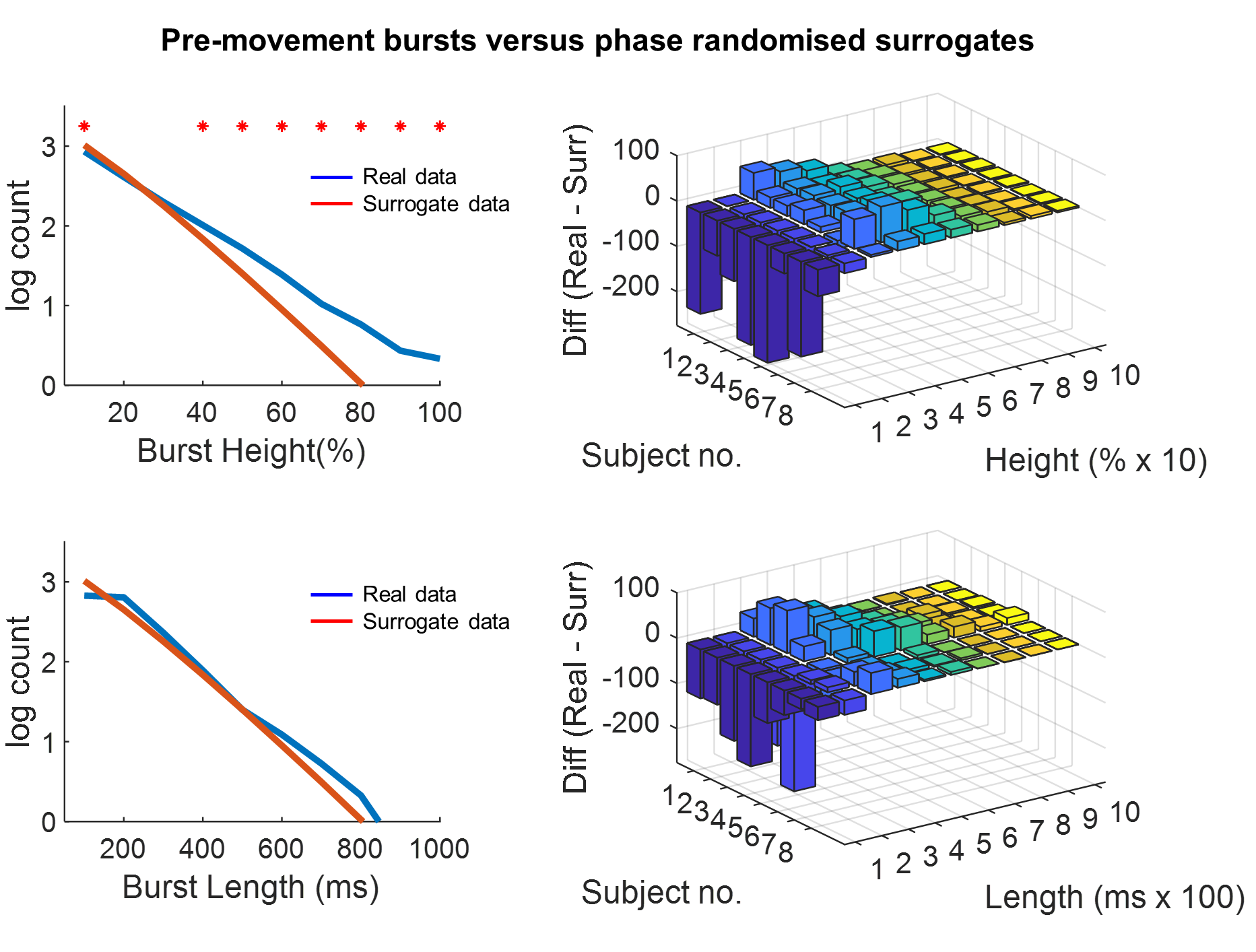

Supplement: S2 Fig — Group-level line histogram of the log10 mean burst counts for different heights (top left) and lengths (bottom left) for real versus surrogate pre-movement data (*p < 0.05, FDR corrected). Difference in burst height and length counts shown for each subject compared with bursts from a surrogate phase-randomised dataset (burst height/length real data minus burst height/length surrogate data; right panels). This demonstrates a relative reduction in the number of the smallest bursts in the real compared with surrogate pre-movement data, with an increase in larger bursts (same analysis shown for post-movement data in Fig 3). The underlying data can be found at https://osf.io/eu6nx/. FDR, false discovery rate. (TIF) [file pbio.3000479.s003.tif]

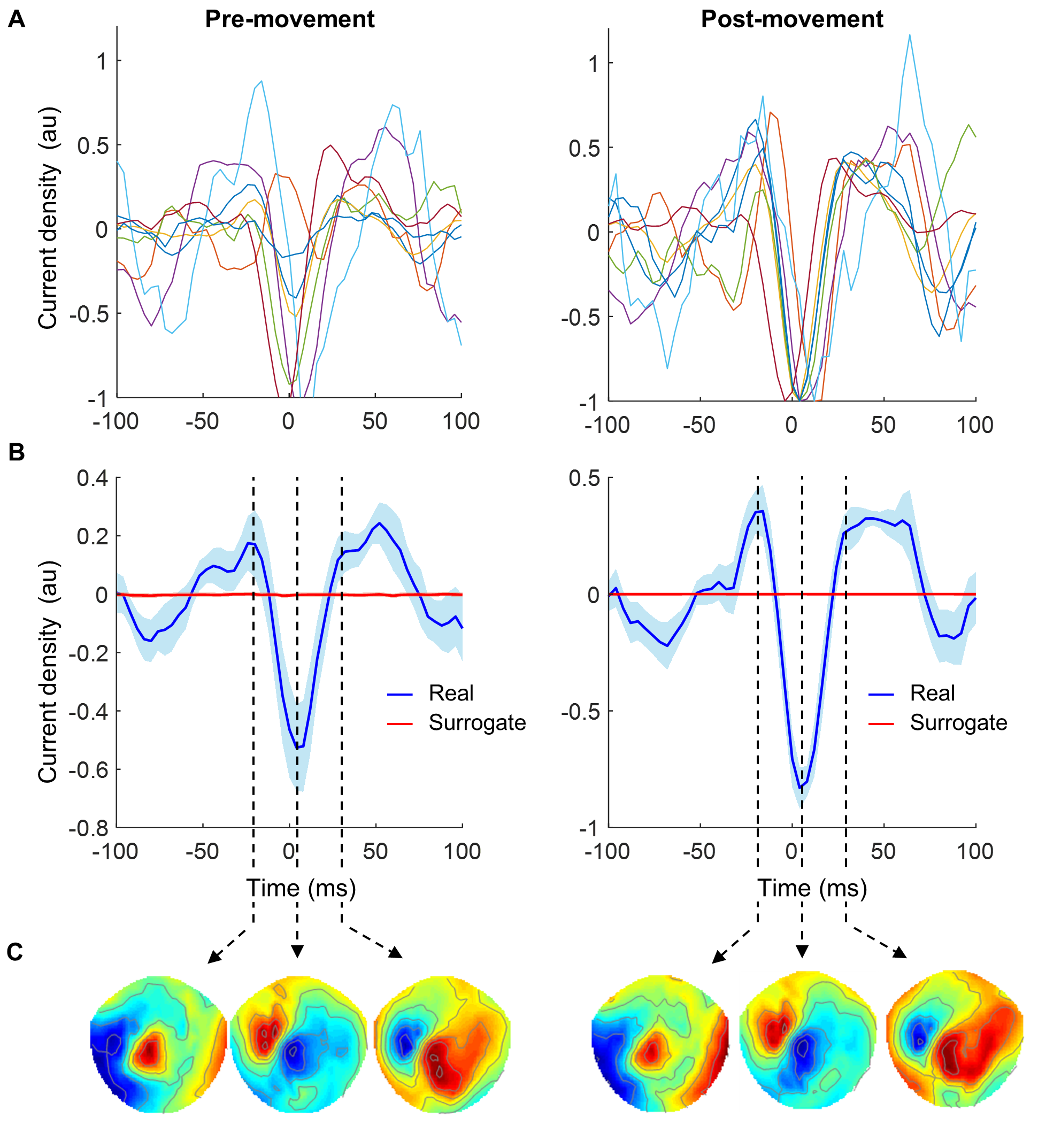

Supplement: S3 Fig — (A) Individual (mean and normalised) bursts from all subjects shown in the time domain, source space (left M1). (B) Mean beta bursts (normalised to average ERS burst maximum) across all subjects for real (blue) versus phase-randomised, surrogate (×1,000 randomisations) data (red). (C) Sensor-level scalp maps (normalised within subjects and averaged over all bursts/subjects) aligned to three periods of maximum extrema (indicated by vertical dashed lines). These show a single dipole at each extrema. Left panel: pre-movement. Right panel: post-movement. The underlying data can be found at https://osf.io/eu6nx/. ERS, event-related synchronisation. (TIF) [file pbio.3000479.s004.tif]

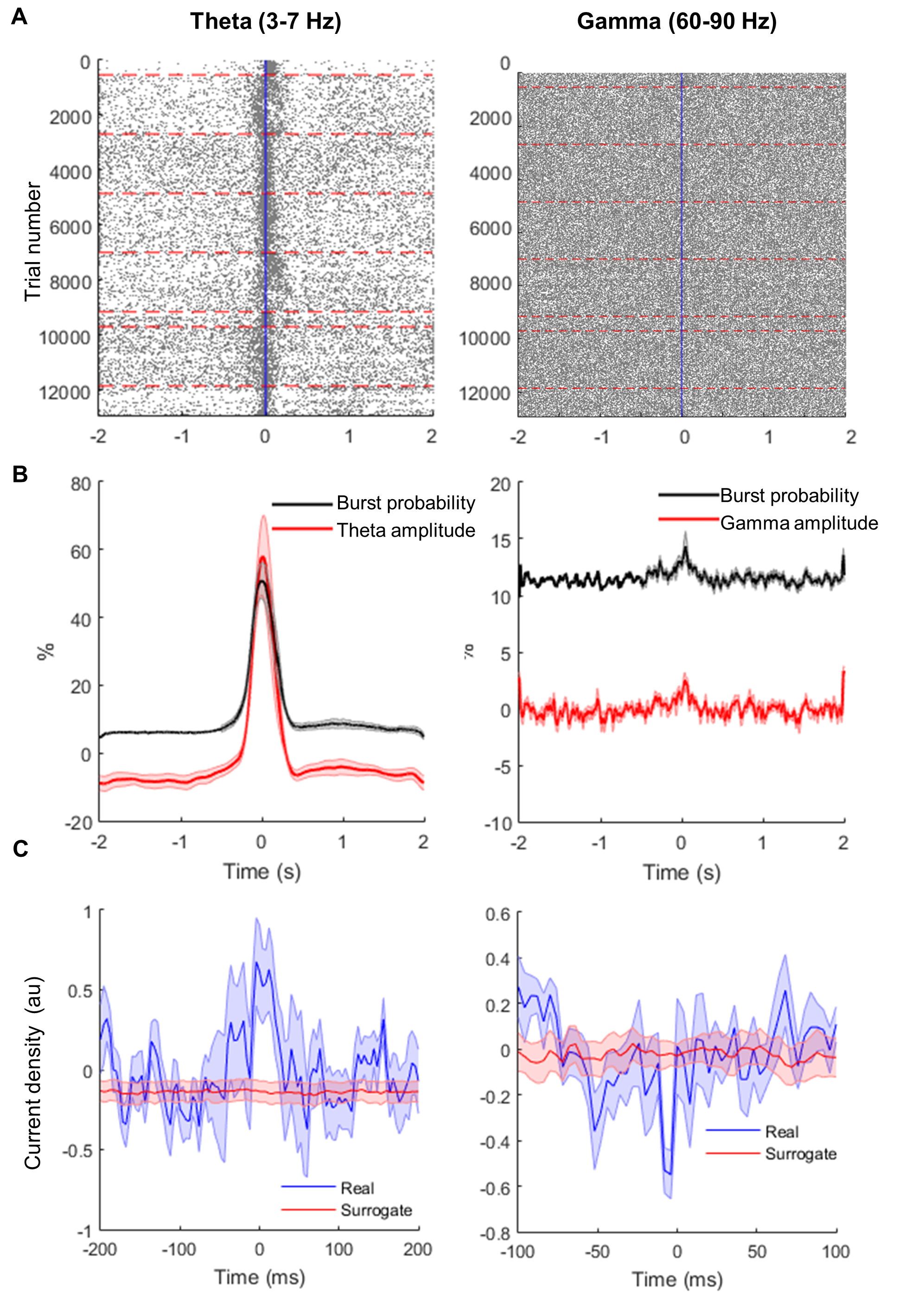

Supplement: S4 Fig — (A) Raster plots for burst distribution according to button press (vertical blue line) for theta (3–7 Hz; left) and gamma (60–90 Hz; right) for all subjects and all trials (individual subjects divided by red dashed line). (B) Amplitude in theta (left) and gamma (right) ranges shown in red, with the probability of bursts in the respective frequency ranges shown in black. The strong burst at button press (0 milliseconds) for theta will include a component of low-frequency movement-related evoked response. There is also a small burst of gamma at movement onset. There was no significant difference found in burst probability according to response time (pre-movement) or decision error (post-movement) for theta or gamma (p > 0.05). (C) Individual average burst shapes in the time domain (centred on the amplitude peak) show different shapes of activity from beta specific bursts. The underlying data can be found at https://osf.io/eu6nx/. (TIF) [file pbio.3000479.s005.tif]
