# Supplementary material for: Human motor cortical beta bursts relate to movement planning and response errors
Source: PLoS Biol. 2019 Oct 4;17(10):e3000479. doi: 10.1371/journal.pbio.3000479 (PMC6795457; doi:10.1371/journal.pbio.3000479)
Supplement: S1 Text — (DOCX) [file pbio.3000479.s008.docx]

**SUPPORTING METHODS**

***Magnetic resonance imaging***

The first anatomical sequence used a 12-channel head coil and was radiofrequency (RF) and gradient spoiled T1 weighted, 3D fast low angle shot (FLASH) sequence with an image resolution of 1mm^3^, field-of-view: 256,256 and 192 mm along the phase (A–P), read (H–F), and partition (R–L) (3T Magnetom TIM Trio, Siemens Healthcare, Erlangen, Germany). Excitation flip angle was set to 12° to ensure sufficient signal-to-noise ratio and repetition time was set to 7.96 ms. MPM protocol images consisted of 3 spoiled multi-echo 3D FLASH acquisitions (800 µm isotropic resolution, proton density, T1 or MT-weighting) using a 32 channel headcoil with two additional sequences used for calibration and correction of inhomogeneities in the radio-frequency transmit field. To achieve an MT-weighting, a Gaussian RF pulse 2 kHz of resonance with 4 ms duration and nominal flip angle of 220° was applied. The field of view was set to 224, 256 and 179 mm along the phase (A-P), read (H-F) and partition (R-L) directions respectively. Alternating readout gradient polarity (Gradient Echoes) were acquired at eight equidistant echo times ranging from 2.34 to 18.44 ms, in 2.30 ms steps (bandwidth of 488 Hz/pixel, 6 echoes for repetition time of 25 ms for all FLASH volumes). Transmit field inhomogeneities were mapped using a 2D STEAM approach, including corrections for geometric distortions of EPI data [1]. Total acquisition scanning time for all protocols was < 30 min.

***Head-cast fabrication***

Anatomical MRI sequences were used to extract scalp surfaces (SPM12) by registering MRI images to a tissue probability map which classified voxels according to tissue makeup (e.g. skull, skin, grey matter etc.). These were then transformed into a surface using the isosurface function in MATLAB (version 2015A, Mathworks, MA, USA). This surface was converted to a standard template library (STL) format with digital outlines of three fiducial coils placed at conventional sites (left / right pre-auricular and nasion). From this digital image, a positive head model was 3D printed (Zcorp, 600 x 540 dots per inch resolution) and this model placed inside a replica dewar, as in our previous studies [1,2]. Liquid resin was then poured between the two surfaces, resulting in a flexible, subject specific head-cast [2].

***Headcast MEG scanning***

One advantage of the head-cast approach is that it permits multiple recording sessions within each subject with the head being in the same position. This allows for recording large scale individual datasets, but because of the lack of movement and low co-registration error, this does not result in accumulative SNR decreases [1,2]. Movement of the fiducials was continuously measured by the CTF system and the standard deviation of movement averaged across the 3 fiducials and calculated separately for the x, y and z directions. Subjects performed 1 - 4 recording sessions on different days, with each session split into 3 separate runs (with short breaks between them). This led to a total number of 1614 ± 763 (mean ± sd) completed trials per subject taken forward for analysis. Data from individual runs within sessions were concatenated and these datasets from each session analysed individually and results averaged across session for each subject. The data was filtered with a 5th order (Butterworth) bandpass filter (2-100 Hz, Notch filter: 50Hz) and downsampled to 250 Hz. Eye blink artefacts were removed using multiple source eye correction [3] and trials with variance > 2.5 sd away from the mean were excluded. Data was epoched from 3.5 s before to 1.5 s after the imperative stimulus (total 5 s) for the analysis of pre-movement neural activity (imperative stimulus-locked) and to 2 s before to 2 s after the button press for post-movement analysis (response locked).

**Source inversion**

Source reconstruction (estimation of current dipole positions and strengths) was performed using SPM12 and an Empirical Bayesian beamformer (with a supplemental analysis for comparison by Minimum norm inversion; S1 Fig) without Hann windowing, using a Nolte single shell model and a frequency of interest of 1 – 90 Hz [4–6]. Data were extracted at each individual vertex as a virtual electrode by multiplying the sensor level data by the weighting matrix (M) between sensors and source from the inversion and the data reduction matrix (U) that specifies the significance of the modes of data that map to the cortex. This estimated time series data was then taken forward for the analysis of beta bursts by selecting the time series from individual vertices closest to the primary motor (M1) cortex which was visually identified for each individual subject with reference to the hand knob [7,8].

In order to obtain the beta amplitude trace, the time series virtual electrode data was filtered using a 4^th^ order (two pass) Butterworth filter with a frequency range of 13 – 30 Hz. The amplitude (envelope of the filtered time series) was derived by taking the modulus of the Hilbert transformation:

S(t) = | H(u(t)) |

Where S(t) is the amplitude signal, u(t) is the filtered data and H represents the Hilbert transform. For time resolved spectrograms (Fig 2A), a Wavelet convolution was implemented (ft_specest_wavelet script in Fieldtrip - Morlet Wavelet, width = 10, gwidth = 5, [9].

**Burst threshold definition**

In order to formally identify beta bursts, previous studies have generally used heuristic thresholds, relative to the mean beta amplitude, but demonstrated robustness of findings across a range of threshold levels [10–12]. This works well for individual studies but limits the scope for comparison across different brain sites and recording methods as well as theoretically subsampling a smaller distribution of relevant bursts (if the threshold is set too high).

Here we adapted recent approaches that correlate burst frequency with total beta power in a trial across a range of thresholds, and define the burst threshold based on the correlation maxima [13]. Specifically, we defined the threshold in terms of standard deviations away from the median signal (as opposed to multiples of the median) of the beta amplitude. In contrast to using the beta power, this approach does not require transformation of the underlying signal. For comparison we repeated the procedure using beta power and found that this resulted in a modestly higher burst threshold definition (S1 Fig 1), as per previous accounts [14]. For each subject we correlated the trial-wise mean beta amplitude with the number of burst events in each trial (defined according to an amplitude threshold), separately for pre- and post-movement periods. Each subject performed 180-540 trials per recording session. Following automated rejection, this may leave, for example, 535 valid trials. In such a case therefore, we would correlate the 535 mean amplitudes (across 13 – 30 Hz) in the pre-movement period against the 535 burst counts in the same pre-movement period (repeated separately for the post- movement period). This procedure resulted in one correlation value for each subject for the pre-movement period and one correlation value for the post-movement period, per burst definition threshold. We then repeated this across a range of thresholds (defined in terms of SDs above the median beta amplitude) to generate two curves for each subject, which showed the relationship between amplitude thresholds used to define the burst and the correlation coefficient between the beta amplitude and burst counts across trials [13]. These curves were then averaged across all subjects and the peak taken at the group level (S1 Fig), as the definition of the amplitude thresholds for defining bursts. This revealed a peak correlation between average beta power and burst amplitude at 1.75 standard deviations above the median, which was consistent for both the pre-movement, imperative stimulus–locked period, and the post-movement, button-locked period (S1 Fig). The threshold of 1.75 SDs above the median was then used for each individual subject, defined according to their own dataset, so that they had statistically matched thresholds, although the absolute threshold levels could differ to take account of varying SNR across subjects. Notably, this threshold was robust to MEG inversion method (S1 Fig).

Comparing our empirically derived threshold with previously used burst definition thresholds, we find that our threshold is higher than in previous subcortical recordings [10,11], but is comparable to findings in the motor cortex in primates [12], and lower than in human sensory cortex [13]. The fact that this threshold level found here is lower than that of other MEG data may partly related to the use of amplitude correlations rather than power correlations [13]. Indeed, using power (as opposed to amplitude) increases the empirically derived threshold, but also leads to flatter correlation curves in our dataset (S1 Fig). Furthermore, the difference in threshold may additionally reflect the difference in recording technique used and the higher SNR datasets obtained with our head-cast MEG approach [1,2,15]. Based on our empirically defined threshold, bursts were identified by locating peaks above this threshold in the beta-filtered amplitude traces from the M1 source-localised data. The 1.75 sd threshold was used to define burst occurrence, and burst duration being the period 1 sd above the median so as not to underestimate the burst length by just examining the central portion of the peaks [13].

**Mixed modelling fit metrics**

In order to compare the predictive power of single trial beta amplitude, burst rate, and burst timing on behaviour, we also fit, for both response time and correctness, separate generalised linear effects models for each measure. These models had the same structure as the full models described previously, but included only single trial beta amplitude, burst rate, or burst timing as fixed effects (in addition to stimulus coherence, congruence, and their interaction). As these models were non-nested, they were compared in terms of relative AIC and BIC values compared to those of the worst model.

We also wanted to determine the fit in terms of variance accounted for. However, variance accounted for is ill-defined for linear and generalized linear mixed models [16]. We did however, calculate the pseudo-R^2^ value for each model as an estimate and to check the model fit which demonstrated the following:

Pre-movement for response time:

Burst time model: R^2^ =0.5399; Burst rate model:  R^2^ =0.5391; Beta amplitude model:  R^2^ =0.5389

Post-movement for error:

Burst time model:  R^2^=0.4063; Burst rate model: R^2^=0.4054:Beta amplitude model:  R^2^=0.4053

This shows a high R^2^ for both pre- and post-movement errors and suggests an overall good fit of the models. However, it is notable that the different models are similar in value and this is not unexpected because (as shown in the main paper) these different parameters are correlated. In addition, however, the pseudo-R^2^ uses the residual variance and doesn't take into account the structure in the error terms (random effects). As with all methods for R^2^ estimation in linear mixed models, this limits generalisation and means all these values should be carefully considered.

**Supplemental References**

1. Troebinger L, López JD, Lutti A, Bradbury D, Bestmann S, Barnes GR. High precision anatomy for MEG. Neuroimage. 2014;86: 583–91. doi:10.1016/j.neuroimage.2013.07.065

2. Meyer SS, Bonaiuto JJ, Lim M, Rossiter H, Waters S, Bradbury D, et al. Flexible head-casts for high spatial precision MEG. J Neurosci Methods. Elsevier B.V.; 2017;276: 38–45. doi:10.1016/j.jneumeth.2016.11.009

3. Berg P, Scherg M. A multiple source approach to the correction of eye artifacts. Electroencephalogr Clin Neurophysiol. 1994;90: 229–41. Available: http://www.ncbi.nlm.nih.gov/pubmed/7511504

4. López JD, Litvak V, Espinosa JJ, Friston KKJ, Barnes GR. Algorithmic procedures for Bayesian MEG/EEG source reconstruction in SPM. Neuroimage. The Authors; 2014;84: 476–487. doi:10.1016/j.neuroimage.2013.09.002

5. Nolte G. The magnetic lead field theorem in the quasi-static approximation and its use for magnetoencephalography forward calculation in realistic volume conductors. Phys Med Biol. 2003;48: 3637–3652. doi:10.1088/0031-9155/48/22/002

6. Belardinelli P, Ortiz E, Barnes GR, Noppeney U, Preissl H. Source reconstruction accuracy of MEG and EEG Bayesian inversion approaches. PLoS One. Public Library of Science; 2012;7: e51985. doi:10.1371/journal.pone.0051985

7. Yousry TA, Schmid UD, Alkadhi H, Schmidt D, Peraud A, Buettner A, et al. Localization of the motor hand area to a knob on the precentral gyrus. A new landmark. Brain. 1997;120 ( Pt 1): 141–57. Available: http://www.ncbi.nlm.nih.gov/pubmed/9055804

8. Dechent P, Frahm J. Functional somatotopy of finger representations in human primary motor cortex. Hum Brain Mapp. 2003;18: 272–283. doi:10.1002/hbm.10084

9. Oostenveld R, Fries P, Maris E, Schoffelen J-M. FieldTrip: Open source software for advanced analysis of MEG, EEG, and invasive electrophysiological data. Comput Intell Neurosci. 2011;2011: 156869. doi:10.1155/2011/156869

10. Tinkhauser G, Pogosyan A, Tan H, Herz D, Kühn AA, Brown P. Beta burst dynamics in Parkinson’s disease OFF and ON dopaminergic medication. Brain. 2017;140: 2968–2981. doi:10.1093/brain/awx252

11. Tinkhauser G, Pogosyan A, Little S, Beudel M, Herz D, Tan H, et al. The modulatory effect of adaptive deep brain stimulation on beta bursts in Parkinson’s disease. Brain. 2017;140: 1053–1067. doi:10.1093/brain/awx010

12. Feingold J, Gibson DJ, DePasquale B, Graybiel AM. Bursts of beta oscillation differentiate postperformance activity in the striatum and motor cortex of monkeys performing movement tasks. Proc Natl Acad Sci. 2015;112: 201517629. doi:10.1073/pnas.1517629112

13. Shin H, Law R, Tsutsui S, Moore CI, Jones SR. The rate of transient beta frequency events predicts behavior across tasks and species. Elife. 2017;6: e29086. doi:10.7554/eLife.29086

14. Sherman MA, Lee S, Law R, Haegens S, Thorn CA, Hämäläinen MS, et al. Neural mechanisms of transient neocortical beta rhythms: Converging evidence from humans, computational modeling, monkeys, and mice. Proc Natl Acad Sci. 2016; 201604135. doi:10.1073/pnas.1604135113

15. Troebinger L, López JD, Lutti A, Bestmann S, Barnes GR. Discrimination of cortical laminae using MEG. Neuroimage. 2014;102: 885–893. doi:10.1016/j.neuroimage.2014.07.015

16. Gaebelein JW, Soderquist DR, Powers WA. A note on variance explained in the mixed analysis of variance models. Psychol Bull. 1976;83: 1110–1112. doi:10.1037/0033-2909.83.6.1110
